# Influence of the intertropical convergence zone on early cretaceous plant distribution in the South Atlantic

**DOI:** 10.1038/s41598-022-16580-x

**Published:** 2022-07-23

**Authors:** Marcelo de A. Carvalho, Cecília C. Lana, Natália P. Sá, Gustavo Santiago, Michelle C. S. Giannerini, Peter Bengtson

**Affiliations:** 1 Laboratorio de Paleoecologia Vegetal (LAPAV), Departamento de Geologia e Paleontologia, Museu Nacional, Universidade Federal do Rio de Janeiro, Rio de Janeiro, 20940-040 Brazil; 2 Institut für Geowissenschaften, Universität Heidelberg, 69120 Heidelberg, Germany

**Keywords:** Palaeoclimate, Palaeoecology

## Abstract

The influence of the Intertropical Convergence Zone (ITCZ) in the emerging South Atlantic region during the late Aptian (Early Cretaceous) is reflected in the spatio-temporal distribution of plant communities recorded in eight Brazilian sedimentary basins. The distribution of the bioclimatic groups of hygrophytes, hydrophytes, tropical lowland flora, upland flora, and xerophytes was quantified using pollen and spores. A predominance of xerophytes from the tropical xerophytic shrubland biome characterized the pre-evaporitic, evaporitic, and post-evaporitic paleoclimatic phases, in particular the evaporitic phase. The region experienced humidity events in the pre-evaporitic and post- evaporitic phases, especially near the paleoequator, where the tropical rainforest biome with two phytophysiognomies (lowland and montane rainforests) prevailed. Increasing humidity had a positive effect on plant diversity.

The Cretaceous was one of the warmest periods of the Phanerozoic and included several greenhouse episodes. The Early Cretaceous Aptian Age (121.4–113.2 Ma)^1^ was characterized by major global changes in climate, physiography, sea level, ocean circulation, and anoxic events^2–7^. Although the late Aptian represents a geologically short time span, the climatic evolution recorded in most Cretaceous sedimentary basins of Brazil can be divided into three phases: pre-evaporitic, evaporitic, and post-evaporitic. In these three phases, warm and dry climates prevailed. The paleoclimatic maps^8–10^ drawn from geological and paleontological evidence indicate a warm, arid belt for low paleolatitudes of South America and Africa during the entire Aptian Age. The recorded flora is typical of warm conditions and is often associated with an arid climate. However, fluctuations occurred, mainly affecting the humidity^6,7,11–13^. According to paleoclimate maps, a humid belt appeared in South America only during the Albian (100.5–113.2 Ma)^8–10^. However, the idea of an equatorial region without humidity is untenable^9^. The humid belt in the paleoequator region is interpreted as analogous to the present Intertropical Convergence Zone (ITCZ)^6,7,9,14,15^. The ITCZ is a relevant meteorological system active in the tropics (approximately 3° N of the equator), where the trade winds of the northern and southern hemispheres come together and rise into the stratosphere, causing increased humidity and consequently, high rainfall in the region^16,17^. Simultaneously with the appearance of the humid belt (ITCZ) in the Aptian or, as a consequence, fully marine conditions were established in the South Atlantic Ocean^18^, a process that created new habitats, affected sedimentation, and altered the climate on a regional and global scale. It is assumed that marine transgressions create more humid climatic conditions, especially in coastal regions. A combination of these factors (the ITCZ and transgression) strongly changed life on land and at sea during the late Aptian.

Climate plays an important role in determining plant distribution and diversity^19–21^. There is ample empirical evidence for the climatic control of plant diversity patterns^22^, especially on large scales^23^. This control is expected to have functioned also in the past.

Numerical climate simulation for the Aptian is sparse. Refer.^24^ using simulations of the Atmospheric General Circulation Model (AGCM) coupled with a 1.5-layer reduced-gravity ocean model for the Aptian, show an increase in precipitation in South America attributed to the ITCZ. According to refer.^24^, the proto-Atlantic Ocean originated from the breakup of Gondwana caused significant changes in rainfall across the earth.

Using experiments of the Fast Ocean–Atmosphere Model (FOAM) for the Aptian of the South Atlantic, refer.^25^ suggested that the central segment of the widening South Atlantic area was affected by strong rainfalls also assigned to ITCZ. A coupling of the dynamic global vegetation model (Lund–Potsdam–Jena dynamic global vegetation model—LPJ), performed within the FOAM for the Aptian, shows a decreasing trend of arid regions, and the Aptian being pivotal with the onset of humid and hot conditions^26^.

The global evidence of the effects of the ITCZ on the biota and sedimentary processes in the South Atlantic is sparse. Here we recognize the influence of the ITCZ in a wide geographical area (central-north, northeastern, and southeastern Brazil) by palynological data, obtained from 79,092 occurrences of terrestrial palynomorphs in 18 sedimentary sections from the Bragança- Viseu, São Luís, Parnaíba, Ceará, Araripe, Potiguar, Sergipe, and Espírito Santo sedimentary basins (Fig. 1 and Supplementary Table 1), from 1 to 20° S, extending from the present equator region to southeastern Brazil (Fig. 1).

**Figure 1:**
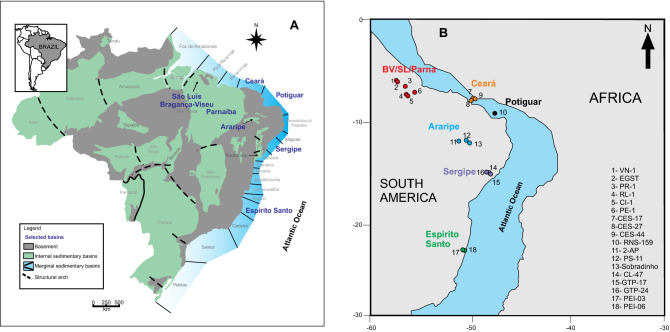
Location of sedimentary basins and stratigraphic sections in Brazil. (**A**) Map showing Brazilian sedimentary basins. The figure was created by Freehand MX (https://www.adobe.com/mena_en/products/freehand/). (**B**) Paleogeographic setting of the late Aptian showing the sites of stratigraphic sections. The Reconstruction map at 116 Ma was generated by ODSN Plate Tectonic Reconstruction Service (https://www.odsn.de/odsn/services/paleomap/paleomap.html).

The late Aptian age of the samples is based on the *Sergipea variverrucata* Biozone recognized in all studied drill cores, which in turn is correlated with part of the upper Aptian *Globigerinelloides algerianus* Zone^27^.

Based on a quantitative approach, we found that the late Aptian palynofloras recorded in the basins show a general predominance of xerophytic plants, attesting to more dry conditions, especially during the deposition of evaporites. However, with a clear humidification trend towards the end of the late Aptian (post-evaporitic phase), predominance was expressed mainly by the quantitative and diversity increases of plant groups: hydrophytes, hygrophytes, tropical lowland flora, and upland flora (e.g., conifers adapted to uplands and humidity).

## Late Aptian climatic evolution in the South Atlantic Ocean

**Figure 2:**
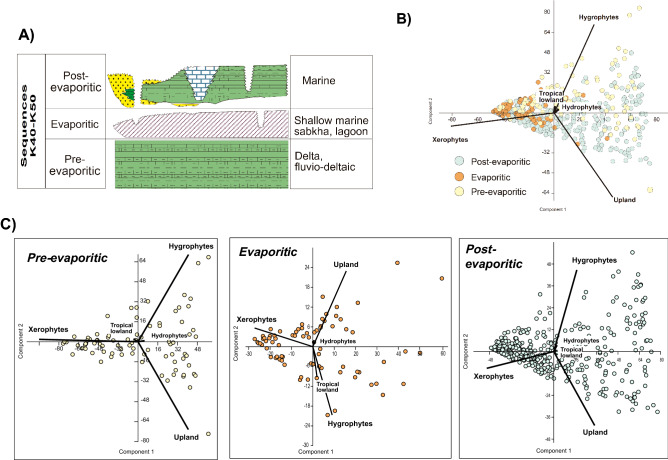
Paleoclimatic phases scheme and principal component analysis for paleoclimatic phases. (**A**) Paleoclimatic phases scheme for the late Aptian and the main depositional environments. (**B**) Principal component plot of bioclimatic groups. (**C**) Principal component for the pre-evaporitic phase (*N* = *92*), evaporitic phase (*N* = *78*), and post-evaporitic phase (*N* = *385*); see Supplementary Fig. 9 for individual basins.

The pre-evaporitic, evaporitic, and post-evaporitic phases are recognized for the late Aptian. These phases are recorded within the K40–K50 sequences (Fig. 2A), and show an average maximum thickness of approximately 650 m in the studied basins. The pre-evaporitic phase is represented by carbonate and siliciclastic deposits formed in fluvial and lacustrine deltaic environments within a large proto-oceanic gulf^28^ (Fig. 2A). The peak of the evaporitic deposition is recorded in the K50 sequence, with widespread occurrences in the Brazilian equatorial margin. The origin of these deposits is the heat intensification associated with the widening of the Atlantic Ocean. These conditions caused strong evaporation leading to a wide distribution of evaporites (mainly halite and anhydrite gypsum) in the South Atlantic basins. The eastern continental margin of Brazil contains a restricted marine section characterized by evaporites, which are particularly prominent in thickness and occurrence in the Espírito Santo Basin (Itaúnas Member of the Mariricu Formation) and the Sergipe Basin (the Ibura Member of the Muribeca Formation)^28^. Evaporites form the most prominent evidence of dry climates in the South Atlantic basins^11^, with evaporation exceeding precipitation. The post-evaporitic phase is characterized by fully marine conditions evidenced by rich assemblages of marine fossils. During this phase, carbonates were deposited, followed by muddy and sandy sediments in shallow-marine and slope environments.

### Paleovegetation

We identified a rich plant community with 139 spore and pollen genera/morphotypes representing all plant groups: bryophytes (five genera), ferns (58 genera), lycophytes (18 genera), pteridosperms (one genus), gymnosperms (27 genera), and angiosperms (30 genera) (Supplementary Table 2). The inferred systematic affinities at the family level reached 100% in bryophytes, 56.9% in ferns, 100% in lycophytes, 100% in pteridosperms, 92.6% in gymnosperms, and 40.0% in angiosperms, totaling 67.6% of the recorded genera (Supplementary Table 2). Marine elements (e.g., dinoflagellate cysts and microforaminiferal linings) were identified, in particular from the Sergipe and Araripe basins (Fig. 1). Pollen grains from gymnosperms were most abundant, represented mainly by the conifer families Cheirolepidiaceae, Araucariaceae, and Podocarpaceae, although representing different climatic settings. *Classopollis* (Cheirolepidiaceae) is the most abundant genus in all sections studied, followed by *Araucariacites* (Araucariaceae). Gymnosperms showed low diversity. Spore-producing plants are the most diverse in the assemblages of all basins (82 genera) and represented by several families of bryophytes, ferns, and lycophytes (e.g., Sphagnaceae, Anemiaceae, Cyatheaceae, Marsileaceae, Selaginellaceae, and Lycopodiaceae). These plant groups depend on water to reproduce and are therefore associated with humid settings.

*Cicatricosisporites* (Anemiaceae) is the third most abundant palynomorph in all the basins, but especially in the northeastern basins (e.g., Sergipe Basin). Angiosperms are among the least abundant; however, they are diverse and include the most abundant and controversial genus *Afropollis*, herein attributed to angiosperms. In the most recent publication that addressed this question, ref.^29^ suggest that *Afropollis* should be treated as an angiosperm genus, although without more precise systematic assignment. The 30 genera/morphotypes of angiosperms are assigned to 8 families, viz., Arecaceae, Chloranthaceae, Euphorbiaceae, Flacourtiaceae, Illiciaceae, Liliaceae, Solanaceae and Trimeniaceae. The second most abundant genus is *Stellatopollis* also without precise systematic assignment.

### Spatio-temporal distribution of bioclimatic groups

**Figure 3:**
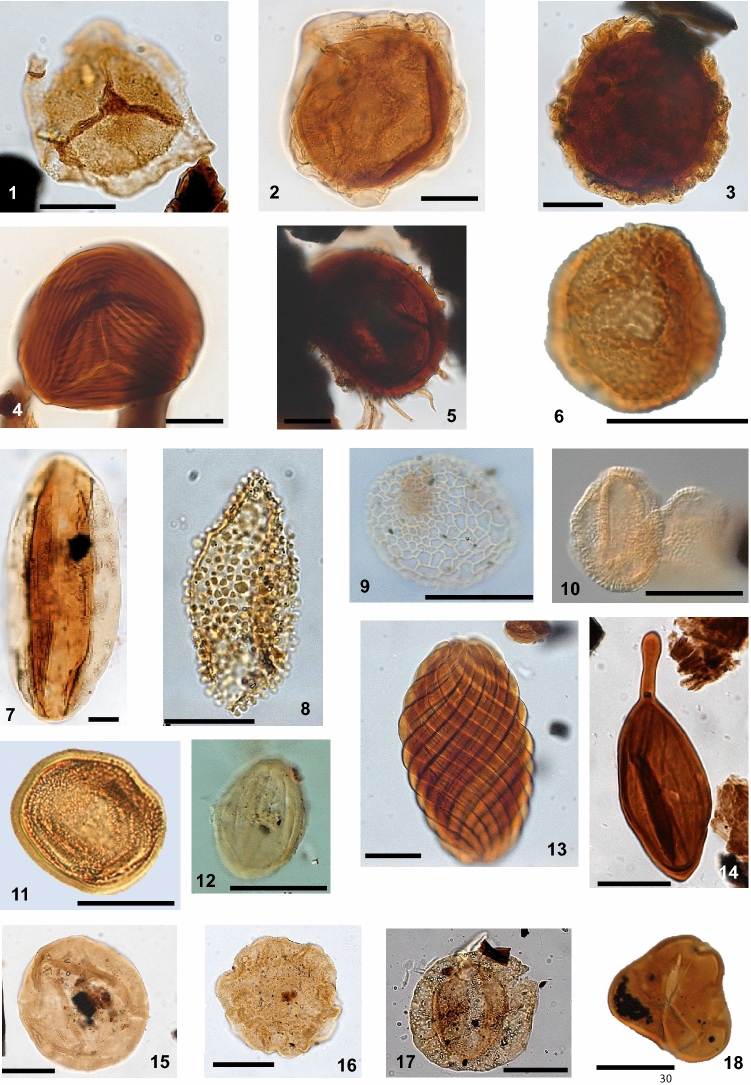
Relevant palynomorphs of bioclimatic groups: (1) *Aequitriradites* sp.; (2) *Crybelosporites* sp.; (3) *Perotriletes* sp.; (4) *Cicatricosisporites* sp.; (5) *Echinatisporis* sp.; (6) *Verrucosisporites* sp.; (7) *Bennettitaepollenites* sp.; (8) *Stellatopollis* sp.; (9) *Afropollis* sp.; (10) *Dejaxpollenites microfoveolatus*; (11) *Classopollis classoides*; (12) *Equisetosporites ovatus*; (13) *Gnetaceaepollenites jansonii*; (14) *Regalipollenites* sp.; (15) *Araucariacites* sp.; (16) *Callialasporites dampieri*; (17) *Complicatissacus cearensis*; (18) *Cyathidites* sp.. Scale bar 20 µm.

On the basis of their botanical affinities, most taxa were classified into five bioclimatic groups [see "Methods" section and Supplementary information], viz., hydrophytes, hygrophytes, tropical lowland flora, upland flora, and xerophytes (Supplementary Table 2) (Fig. 3).

Overall, the vegetation is dominated by the xerophytic bioclimatic group on account of the very high abundance of *Classopollis* (Cheirolepidiaceae) (general mean of 60.5%). However, the stratigraphic distribution of the bioclimatic groups in the sections studied (Supplementary Figs. 1–6) indicates wet phases confirmed by the curves of the other bioclimatic groups (hygrophytes, hydrophytes, tropical lowland flora, and upland flora). We used Pearson correlation analysis (Supplementary Fig. 7) to assess the correlation between the bioclimatic groups. The analysis revealed positive correlations between the bioclimatic groups of hygrophytes, hydrophytes, tropical lowland flora, and upland flora, and a negative correlation between these groups and the xerophyte group (Supplementary Fig. 7). The positive correlation between upland flora and hygrophytes confirms previous studies for the Sergipe Basin^6,7^, suggesting a relation between these groups and the hot and humid climate. The weak negative correlation between tropical lowland flora and upland flora is presumably related to elevation.

The upland flora forms the second most abundant bioclimatic group, with an average of 18.9%. The large number of specimens of *Araucariacites* (Araucariaceae) in this group is notable. The hydrophytes are the least abundant group, with an average of only 1.4%. In this group, the highest values are attributed to the genus *Crybelosporites* (Marsileaceae).

Principal component analyses (PCA) were used to reduce the multidimensional dataset, based on the percent abundance of the bioclimatic groups to a smaller number of dimensions for interpretive analysis. For all sections, two components or axes explain 97.6% of the observed variability (Fig. 2B). Hygrophytes, hydrophytes, tropical lowland flora, and upland flora show positive correlation (positive loading, 0.320, 0.029, 0.006, and 0.468, respectively), whereas xerophytes show a negative relationship (negative loading, − 0.823) on the first axis, which alone explains 83.0% of the variability. In summary, the first axis of the PCA reveals a separation of two major climatic conditions (wet and dry) along the axis (Fig. 2B). The wet conditions include the associations of hygrophytes, hydrophytes, tropical lowland flora, and upland flora, with dry conditions associated with taxa from the xerophyte group. The second axis explains 14.6%, in which hygrophytes, hydrophytes, and tropical lowland flora show a positive correlation relationship (positive loading, 0.719, 0.037, 0.036, respectively), whereas upland flora and xerophytes show a negative relationship (negative loading, − 0.684 and − 0.108, respectively). With respect to the second axis, a polarization between the hygrophytes (positive loading, 0.719) and the upland flora (negative loading, − 0.684) can be interpreted as a lowland–upland trend. The same pattern was recorded for all paleoclimatic phases (Fig. 2C) and sections (Supplementary Fig. 8), that is, the first axis is related to humidity vs. aridity, and the second axis to elevation (lowland vs. upland). This suggests that these two factors, particularly the first one, controlled the vegetation distribution in the late Aptian of the region. As all bioclimatic groups occurred in the three evaporitic phases, these trends in abundance reflect expansion and contraction of the recorded vegetation.

Parallel increasing trends of bioclimatic groups mark the pre-evaporitic phase: hygrophytes and upland flora in the Bragança-Viseu, São Luís, Parnaíba, Ceará, Potiguar, and Araripe basins (Supplementary Figs. 1–3 and 5), suggesting that there was a certain amount of moisture in these areas. The xerophytes show the lowest average of this phase (44.1%) (Table 1), whereas hygrophytes show the highest average (27.0%). These humid conditions are confirmed by the highest mean of the Fs/X ratio (Fs/X = 0.4), representing the predominance of spore-producing plants [see Methods section and Supplementary information]. Despite the low abundance of hydrophytes in the sections, a prominent feature is the highest average (2.5%) of this group (Table 1), which is assigned to aquatic environments, confirming relatively wet conditions in this phase. There are no pre-evaporitic samples available from the Sergipe and Espírito Santo basins.

**Table 1:** Average abundance of bioclimatic groups, diversity, Fs/X and marine elements for the paleoclimatic phases.

Paleoclimatic Phases	Hygrophytes	Hydrophytes	Tropical lowland flora	Upland flora	Xerophytes	Diversity (H’)	Fs/X	Marine
Pre-evaporitic	**27.0**	**2.5**	**4.0**	**22.5**	44.1	**1.8**	**0.4**	0.2
Evaporitic	8.3	0.5	3.3	11.6	**76.4**	1.2	0.1	3.9
Post-evaporitic	11.6	1.2	2.0	**24.4**	60.9	1.3	0.2	**44.1**
General average	15.6	1.4	3.1	19.5	60.5	1.4	0.3	16.1

In bold, the most significant values. Numbers of bioclimatic groups (hygrophytes, hydrophytes, tropical lowland flora, upland flora and xerophytes) in average percentage. Numbers of marine elements in average absolute abundance.

The evaporitic phase is characterized by the highest abundance of the xerophyte bioclimatic group (76.4%) (Table 1), represented mainly by *Classopollis* (Supplementary Figs. 1–6). A high abundance of xerophytes occurred widely distributed in all basins studied. In this phase, tropical lowland flora is notable, showing an average higher than the overall average (3.3%), particularly in the Bragança-Viseu, São Luís, Parnaíba, and Ceará basins (Supplementary Figs. 1 and 2). This result is related to the moderate to high abundance of the genus *Afropollis* in these basins. The evaporitic phase is also characterized by the lowest average Fs/X ratio (Fs/X = 0.1) (Table 1), confirming the dominance of xerophytes.

The post-evaporitic phase is characterized by the upland flora bioclimatic group (mean = 24.4%) (Table 1). The moderate to high abundance of upland flora in this phase is represented, in particular, by pollen grains of *Araucariacites*, which represent the high-relief family Araucariaceae. This bioclimatic group is associated with more humid conditions, as confirmed by an Fs/X ratio higher than the overall average (Fs/X = 0.2). The upland flora is significant in all basins, except the Espírito Santo Basin, where xerophytes predominate in both studied phases in this basin.

### Latitudinal biome distributions

**Figure 4:**
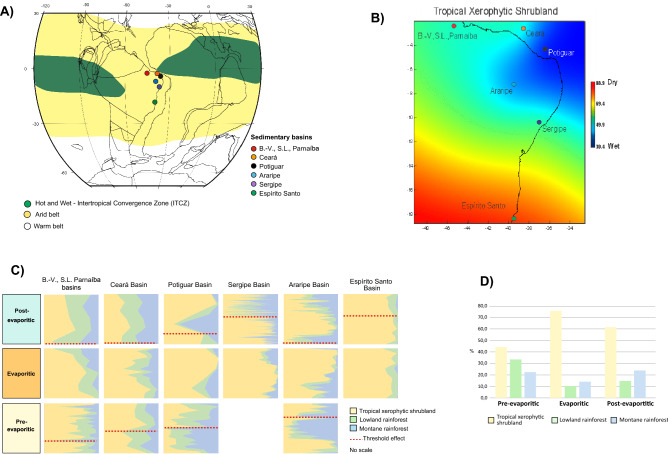
Latitudinal changes in late Aptian biomes from southeast to center-north. (**A**) Paleoclimatic belts of the late Aptian in South America (climatic belts modified from refer.^14^). Reconstruction map at 116 Ma modified from ODSN Plate Tectonic Reconstruction Service. The Reconstruction map at 116 Ma was generated by ODSN Plate Tectonic Reconstruction Service (https://www.odsn.de/odsn/services/paleomap/paleomap.html). (**B**) Late Aptian latitudinal distribution of the tropical xerophytic biome in Brazil. (**C**) Stratigraphic distribution of biomes for individual basins. (**D**) Relative Importance of biomes for paleoclimatic phases.

**Figure 5:**
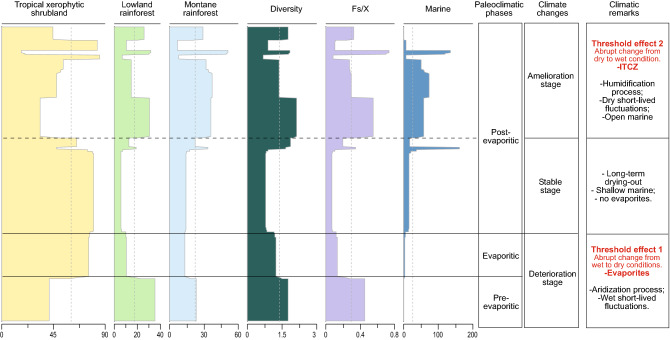
Biome trends in relation to paleoclimatic phases. Change in biomes, diversity, Fs/X ratio and marine elements shown by changepoint analysis plotted against paleoclimatic phases.

Biome change is a fundamental biological response to climate change. In the study area, the predominance of a specific biome is mainly related to humidity, since all five recorded bioclimatic groups are related to a warm climate (Supplementary Table 2) representing two biomes: tropical xerophytic shrubland and tropical rainforest. In the rainforest biome two phytophysiognomies are recognized: lowland and montane rainforest. The tropical xerophytic shrubland biome predominates in the three paleoclimatic phases, with a wide latitudinal range from the Bragança-Viseu, São Luís, and Parnaíba basins (1° S) to the Espírito Santo Basin (20° S). This wide distribution is compatible with a predominantly arid climate in South America in the late Aptian, as indicated by paleoclimatic maps^8,9,15^ (Fig. 4A). Most arid and semi-arid ecosystems are mainly controlled by precipitation. Other climate parameters are less important, a condition that simplifies cause-effect interpretations. The PCA (Fig. 2B) demonstrated that the wet–dry trend, which reflects high–low precipitation, was the main determinant in the distribution of the biomes. However, considering all phases, an increasing trend in humidity was observed from the southeast (Espírito Santo Basin) to the northeast (e.g., Potiguar Basin) (Fig. 4B), coinciding with the hot and wet belt attributed to the ITCZ (Fig. 4A)^15^. The latitudinal distribution of diversity also follows this trend. Diversity increased significantly towards in the basins near the equator. Diversity indices (Shannon – H’) peaked in the Sergipe Basin (H’ = 3.5, CL-47 section) at 11° S. Conversely, the lowest average diversity is recorded in the Espírito Santo Basin (H' = 1.1) at 20° S. Additionally, there is a clear correlation between high diversity (H') and humidity (Fs/X ratio) (r = 0.691), regardless of paleoclimatic phase, as evidenced by the synchronicity of the H' and Fs/X curves (Fig. 5). After data normalization between humidity (Fs/X) and marine elements (dinoflagellate cysts and microforaminifer linings), we performed linear correlation analyses, which showed a weak but positive correlation (r = 0.137). This is due to the fact that pre- evaporitic deposits contain only 19 occurrences of dinoflagellate cysts in 90 samples. Despite this, the curves of Fs/X, marine elements and diversity are synchronous (Fig. 5), suggesting a relation between humidity, diversity, and marine incursions.

The pre-evaporitic phase is marked by a certain balance between the biomes (Fig. 4C,D). In the lowlands, the tropical xerophytic shrubland biome predominated in the Bragança- Viseu, São Luís, Parnaíba, and Ceará basins, but in the Potiguar Basin it is co-dominant with the lowland rainforest. The montane rainforest was relatively extensive in this phase, although with several areal changes, and reached its widest extent in the Araripe (7° S) and Potiguar (5° S) basins in response to the deterioration of the tropical xerophytic shrubland biome. These conditions demonstrate that humidity was relatively high at this stage. The pre-evaporitic deposits were characterized by the highest diversity average (H’ = 1.8).

The method of indicator species analysis (IndVal) was used to identify the key species of each paleoclimatic phase (Supplementary Table 15). The species identified for the pre-evaporitic phase, *Deltoidospora* spp. (Cyatheaceae-Dicksoniaceae) related to the montane rainforest, are indicator species for the Bragança-Viseu, São Luís, Parnaíba, and Ceará basins. The *Gnetaceaepollenites* spp. (Gnetaceae) of the Potiguar Basin and *Equisetosporites* spp. (Ephedraceae) of the Araripe Basin are related to the tropical xerophytic shrubland biome (Supplementary Table 15). Even for the pre-evaporitic phase, a progressive increase in the tropical xerophytic shrubland biome was observed and interpreted as the start of a climatic deterioration stage (Fig. 4C), which culminated in the evaporitic phase. Shifts in vegetation types may occur when precipitation reaches a threshold value, which means that a regionally synchronous gradual climate change can cause abrupt vegetation shifts. The change from humid to warm and arid conditions (evaporitic phase) is directly related to a decrease in precipitation. This aridization process coincides with the appearance of marine elements (e.g., dinoflagellate cysts). The threshold effect (intense evaporation) is reflected in an abrupt decrease in the abundance of lowland and montane rainforest and a sharp increase to a very high abundance of the tropical xerophytic shrubland biome (Supplementary Figs. 4C and 5). The threshold effect was not detected in the Espírito Santo Basin, where the arid conditions remained stable with minimal shift (expansion and contraction) of the biome. The main representatives of this biome are conifers of the family Cheirolepidiaceae (*Classopollis*), which were most abundant in lagoons and coastal environments and are often associated with evaporates^30–35^. Even under xeric or water-stressed conditions there was a slight increase in biomes related to a humid climate (lowland and montane rainforest phytophysiognomies) towards the equatorial region, suggesting influence of the ITCZ (Fig. 4A,B).

The evaporitic phase was characterized by the lowest diversity average (H’ = 1.2). With modest rainfall, arid regions are generally characterized by fewer species than moister biomes^36^. However, diversity indices peaked in the Bragança-Viseu, São Luís, and Parnaíba basins (H’ = 2.6, RL-01 section) and along the equatorial margin (2° S) (Supplementary Fig. 1).

IndVal emphasizes the xeric conditions in the evaporitic phase by association with the species from the tropical xerophytic shrubland biome: *Classopollis* spp. (Ceará and Potiguar basins), *Classopollis classoides* (Sergipe Basin), *Classopollis intrareticulatus* (Araripe Basin), and *Gnetaceaepollenites* spp. (Espírito Santo Basin). For the Bragança-Viseu, São Luís, Parnaíba, and Ceará basins, where xeric restrictions are milder, the indicator taxon is *Afropollis* spp. from the lowland rainforest. This genus shows the weakest negative correlation with xerophytes.

After the end of evaporite deposition, all sections indicate climatic stability, which kept the climate hot and arid even in the post-evaporitic phase, although the response was not linear.

**Figure 6:**
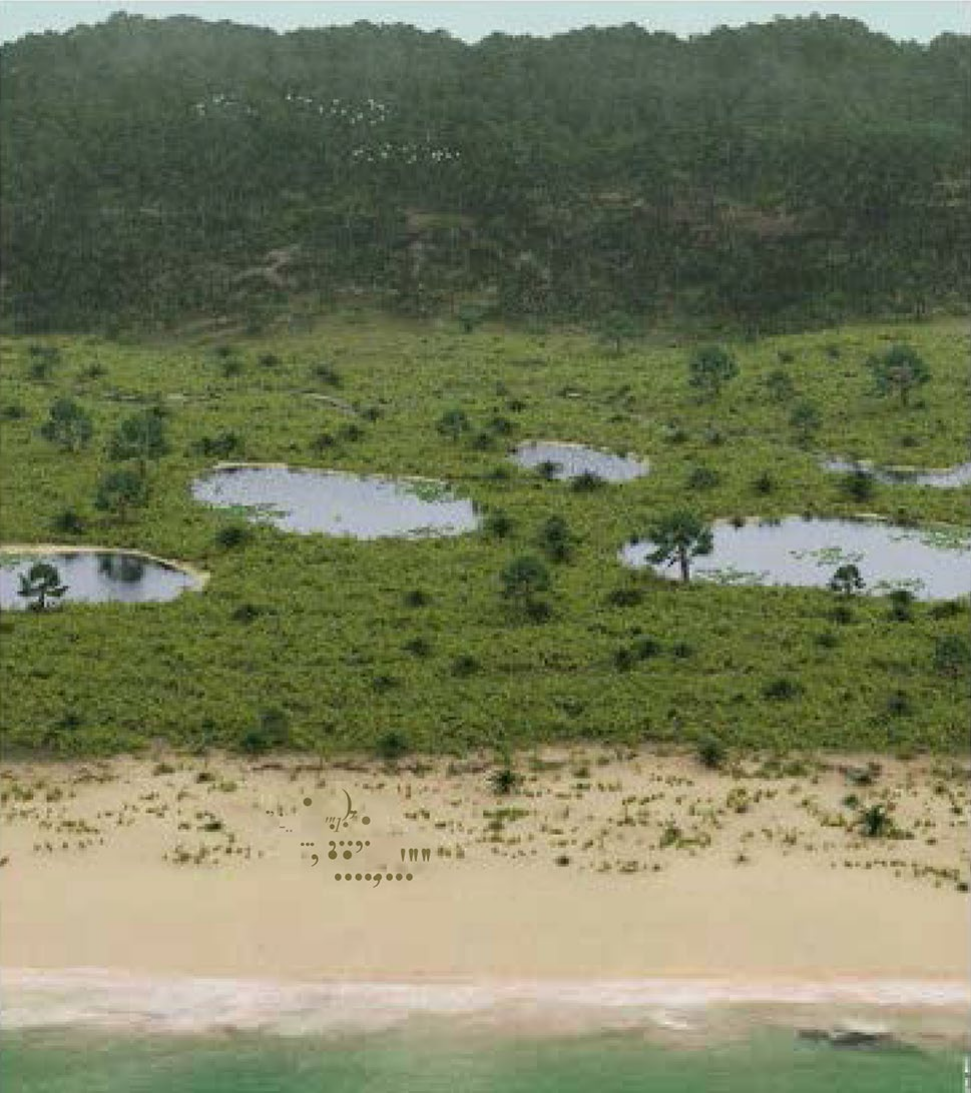
Reconstruction of the transitional gradient between marine to terrestrial environment (uplands) under ITCZ influence. The illustration is based on paleoflora and environmental information from palynological data from studied sections. Original size illustration: 18 × 24 cm, by Julio Lacerda.

The shift in the biomes, especially the tropical xerophytic shrubland in the Bragança-Viseu, São Luís, Parnaíba, Ceará, and Araripe basins, occurred in the transition between the evaporitic and post-evaporitic phases, whereas in the Potiguar and Sergipe basins it occurred within the post-evaporitic phase. As indicated in the dendrograms of each section (Supplementary Figs. 1–6), the shift occurred abruptly in all basins, except the Espírito Santo Basin. The tropical rainforest biome (lowland and montane rainforests) replaced the tropical xerophytic shrubland in almost all basins (Fig. 4C). Even the Espírito Santo Basin, far from the influence of the ITCZ, shows a slight increase in lowland rainforest. The changes in the biomes are attributable to threshold effects caused by gradual climate change related to the ITCZ intensification shift and progressive increase in marine influence, indicated by an increase in marine microplankton from an average of 3.9% in the evaporitic phase to 44.1%. The increase in marine influence is reflected in the first major flooding surface observed in the Cretaceous succession^27^. Thus, a climate amelioration stage was established in the post- evaporitic phase (Fig. 5). In combination with published paleotopographic information^25^, the bioclimatic groups associated to the humid conditions (hygrophytes, hydrophytes, tropical lowland flora, and upland flora) were combined and visualized to create Fig. 6.

According to refs.^7,37^, arid conditions are characterized by sea-level lowstands, whereas warm and humid conditions are correlated with sea levels rise, which explains the increase in the tropical rainforest biome (lowland and montane rainforests). The more intense humidity is supported by the results of IndVal for the post-evaporitic phase, with all species related to humid climate: *Deltoidospora* spp. (Bragança-Viseu, São Luís and Parnaíba basins), *Araucariacites limbatus* (Ceará Basin), *Cicatricosisporites* spp. (Potiguar Basin), *Cicatricosisporites* spp. and *Araucariacites australis* (Sergipe Basin), *Inaperturopollenites* spp. (Araripe Basin) and *Inaperturopollenites simplex* (Espírito Santo Basin).

Our results show that the ITCZ combined with the opening of the South Atlantic Ocean during the late Aptian altered vegetation dynamics. As today, the ITCZ influence is stronger in the northeastern and north-central regions of South America. It is notable that the late Aptian climate evolution in the South Atlantic, culminating in higher humidity, was accompanied by an intrinsic relation between plant diversity, humidity, and marine influence.

## Methods

### Palynology

Of the 555 samples, 479 were prepared at the Research and Development Center of Petrobras (CENPES), 38 samples of well PS-11^38^ were prepared at the Palynofacies and Organic Facies Laboratory, Department of Geology of the Federal University of Rio de Janeiro, and 38 samples from the Sobradinho outcrop^39^ were prepared at the laboratories of the São Paulo State University, IGCE - Campus of Rio Claro (UNESPetro - Centro de Geociências Aplicadas ao Petróleo). Preparations were based on the standard method compiled by^40^, based on methods developed by^41–43^, among others. In this method, all mineral constituents are destroyed by hydrochloric and hydrofluoric acids before heavy-liquid separation. The remaining organic matter was sieved through a 10 μm mesh and mounted on permanent slides. The samples were analyzed under a transmitted-light microscope, and the analysis was based on the first 200 palynomorphs counted on each slide. Specimens were analyzed and photographed with a camera mounted on a Zeiss Axio Imager A1 transmitted-light microscope.

### Bioclimatic analysis

The bioclimatic analysis was based on living representatives of the taxa, which provided valuable information about vegetation history over temporal and spatial scales. The recognition of spores and pollen grains as climatic indicators (bioclimatic groups) is supported by botanical affinities (Supplementary Table 2) (Fig. 3). However, the parent plant of a sporomorph is often unknown. Therefore, sporomorph taxa have been classified at the family level. In this regard, we followed the criteria shown by^44,45^: (1) sufficient representation in a number of species in the living world; (2) more or less restricted bioclimatic characteristics; (3) the possibility of identifying a modern climatic optimum in which there is maximum development of the group in terms of number of species and specimens. In this study, the botanical affinities of the indicator species follow previous studies^46–64^. Associated with the concept of botanical affinity, we consider environmentally significant plant categories, which reflect broad co-existing plant communities proposed by^65,66^.

Based on botanical affinities and paleoenvironmental conditions, five bioclimatic groups are proposed: hygrophytes, hydrophytes, tropical lowland flora, upland flora, and xerophytes.

### The biomisation approach

We based our analysis on the biomisation approach^67^ for reconstructed biomes. The basis of the technique is the allocation of pollen taxa to a range of plant functional types (PFTs) and to record how these combine to form biomes^68,69^. In our study, we used the bioclimatic groups as PFTs. Although this technique is used mainly for the Quaternary, the recognition of 67.6% of the botanical affinities supports the application for Cretaceous times.

The bioclimatic groups were assigned to two biomes based on the refers.^10,69^, which denote two main environmental gradients, humidity and elevation: (1) Tropical xerophytic shrubland, characterized by xeromorphic characteristics, particularly fire tolerance. For example, microphyll leaves, thorns, deciduous leaves, thick bark, and stomata are often present. Drought-adapted taxa are common, with dense undergrowth of shrubs and herbs. (2) Tropical rainforest constituting a mix of mesophyllous and sclerophyllous taxa and depending on moisture demand and the length of the dry season, factors that determine the number of deciduous taxa. Tropical rainforests may form belts along tropical coastal areas. In this biome, two phytophysiognomies are recognized: a typical rainforest formation that occurs in lowlands, possibly close to rivers and lakes, and one distributed in higher regions, herein named montane rainforest, with a mixture of mesophyllous and sclerophyllous taxa, inhabiting hills, highs or even mountains, with abundant rainfall and relatively low temperatures. Tree ferns and palms are locally common.

### Biodiversity

Shannon diversity indices (H') for palynomorphs were calculated for all samples using PAST software^70^ (Supplementary Tables 3–8, Supplementary Figs. 1–6). The Shannon Index (often cited as the Shannon-Weaver Index or Shannon-Wiener Index^71^ is sensitive to the total number of species and their relative abundance. The index is calculated by the equation ${\mathrm {H}} ={\text{-}}\Sigma[(\text{pi})^ * \log(\text{pi})]$, where *pi* is the proportion of individual of the *i*-th species. The *pi* is calculated by n/N, where *n* is the individuals of a given type/species, and *N* is the total number of individuals in a community. The diversity index allows a comparison of the palynomorph assemblage in relation to climate phases.

### Fern spores and xerophytes (wet-dry trend)

The co-occurrence of fern spores and xerophytic palynomorphs (*Classopollis* and polyplicate gnetalean pollen) was used to indicate dry-wet trends. The ratio of fern spores to xerophytic palynomorphs (Fs/X) was calculated as Fs/X= nFs/(nFs + nX), where N is the number of specimens counted, Fs fern spores (non- reworked), and X xerophytic pollen grains (Supplementary Tables S3–S8, Supplementary Figs. 1–6). In summary, Fs/X approaching 1 implies high humidity and when approaching − 1 indicates low humidity.

### Marine elements

Marine elements were considered in this study because they attest to the emerging South Atlantic. The progressive transgression revealed by different types of dinoflagellate communities culminates in an open-marine environment in the central segment^25^ of the widening South Atlantic. These elements are composed of dinoflagellate cysts and microforaminiferal linings and counted separately (Supplementary Tables 3–8, Supplementary Figs. 1–6).

### Pearson correlation analysis

The Pearson analysis (+/− 1) obtained from the percentage abundance of bioclimatic groups was used to yield a correlation matrix and identify the relationships of the groups. This coefficient reflects the presence or absence of similarity among the taxa. A coefficient approaching 1 implies a positive correlation, and − 1 a negative correlation among the bioclimatic groups.

The Pearson correlation shows an overall positive correlation between hydrophytes, hygrophytes, tropical lowland flora, and upland flora, and negative correlations among these groups and xerophytes (Supplementary Fig. 7).

### Principal components analysis (PCA)

We performed PCA using PAST software^70^ on individual samples. PCA is a mathematical technique for simplifying a multidimensional dataset (here, five bioclimatic groups: hygrophytes, hydrophytes, tropical lowland flora, upland flora, and xerophytes). This technique is helpful in a multivariate analysis to structure and visualize larger datasets by reducing a large number of variables to a few linear combinations (principal components). It was used to delineate the main factors that influenced the vegetation distribution in the climatic phases (pre-evaporitic, evaporitic, and post- evaporitic) of the general dataset (Fig. 2B,C) and for each basin (Supplementary Fig. 8).

### Indicator species analysis (IndVal)

IndVal was introduced by^72^ to determine whether species are characteristic of particular sample groups. The technique has been applied recently to fossil material^6,72–75^ and can provide evidence of alterations in the biota, paleoenvironment, paleoclimate, and paleoceanography. In this study, the paleoclimatic phases (pre-evaporitic, evaporitic, and post-evaporitic phases) were used as sample groups. IndVal is determined by the formula IndVal Group k, species j = 100 × Ak, j × Bk, j, where Ak, j = specificity and Bk, j = fidelity, and the values were obtained using PAST software^70^. The results are presented as percentages (Supplementary Table 15). The indicator species analyses are strongly associated with particular climatic phases in each basin studied, even those with low abundances (for example, *Deltoidospora* spp.) and more abundant species, such as *Classopollis classoides* and *Araucariacites australis*. Indicator species for the pre-evaporitic phase of all basins are related to plants of the upland flora (*Deltoidospora* spp.) and xerophytes (*Gnetaceaepollenites* spp. and *Equisetosporites* spp.). The evaporitic phase is dominated by xerophytes (*Classopollis*), and the post-evaporitic phase is represented by *Inaperturopollenites* spp. and *Cicatricosisporites* spp..

### Changepoint analysis

We performed changepoint analysis using PAST software^70^ on individual samples. The method is used to detect subtle changes in the data series^76^. The analysis was based on the bioclimatic groups into three biomes (tropical seasonal forest, moist montane forest, tropical xerophytic shrubland), diversity index, Fs/X ratio, and marine elements (Fig. 5).

## Supplementary Information

Supplementary Information.

## Data Availability

The data and code used in this paper are deposited at CENPES, PETROBRAS, Rio de Janeiro, RJ, Brazil (wells VN-1, EGST-1, RL-1, PE-1, CI-1, PR-1, CL-47, CES-17, CES-27, CES-44, RNS-159, 2-AP, PEI-03, and PEI-06); the laboratory LABMICRO of the Department of Geology, Institute of Geoscience, Federal University of Rio de Janeiro, Rio de Janeiro, Brazil (well PS-11) and the São Paulo State University (UNESP) collection, IGCE-UNESPetro, in Rio Claro, SP, Brazil (Sobradinho section). Additional information on samples (wells VN-1, EGST-1, RL-1, PE-1, CI-1, PR-1, CL-47, CES-17, CES-27, CES-44, RNS-159, 2-AP, PEI-03 and PEI-06) can be accessed in www.anp.gov.br.
