# Supplementary material for: Influence of the intertropical convergence zone on early cretaceous plant distribution in the South Atlantic
Source: Sci Rep. 2022 Jul 23;12:12600. doi: 10.1038/s41598-022-16580-x (PMC9308796; doi:10.1038/s41598-022-16580-x)
Supplement: Supplementary file 1 — Supplementary Information. [file 41598_2022_16580_MOESM1_ESM.pdf]

Supplementary information for

Influence of the intertropical convergence zone on early Cretaceous plant distribution in the South Atlantic

Marcelo de A. Carvalho[1]*, Cecília C. Lana[1], Natália de P. Sá[1], Gustavo Santiago[1], Michelle C. S. Giannerini[1], Peter Bengtson[2]

[1]Laboratorio de Paleoecologia Vegetal (LAPAV), Departamento de Geologia e Paleontologia, Museu Nacional, Universidade Federal do Rio de Janeiro; 20940-040, Rio de Janeiro, Brazil.

[2]Institut für Geowissenschaften, Universität Heidelberg, 69120, Heidelberg, Germany.

*Corresponding author. Email: mcarvalho@mn.ufrj.br

This PDF file includes:

Materials
Supplementary Text
References
Figs. S1 to S8
Tables S1 to S15

Materials

<u>Sedimentary basins</u>

According to refer.[28], the Brazilian sedimentary basins are grouped into four large assemblies based mainly on the tectonic context in which they developed: Paleozoic Syneclises (Parnaíba Basin), Meso-Cenozoic extensional margin (Araripe, Sergipe, and Espírito Santo basins), Meso-Cenozoic Transform Margin (Ceará and Potiguar basins), and Mesozoic aborted rift basins (Bragança-Viseu and São Luís basins). The evolution of the sedimentary basins is recognized by several depositional sequences.

The Bragança-Viseu, Ceará, Espírito Santo, Potiguar, São Luis, and Sergipe basins belong to the class of sedimentary basins characteristic of passive continental margins and formed as a result of the South American and African separation. The interior basins (Araripe and Parnaíba basins) are directly linked to the extensional deformation accompanying the opening

of the southern and equatorial branches of the South Atlantic rift system during the Early Cretaceous[77].

Bragança-Viseu, São Luís, and Parnaíba basins

The Bragança-Viseu, São Luís, and Parnaíba basins are treated together, as they present a similar stratigraphic evolution. The Bragança-Viseu and São Luís basins are located on the equatorial margin and the Parnaíba basin in north-central Brazil (Fig. 1). The basins constitute a rift system (graben and semi-graben) located between the terrains of the folding belt. Together, they cover an area of approximately 645,000 km^2. The sedimentary succession of the basins consist of Paleozoic, Mesozoic, and Cenozoic rocks. The Cretaceous strata are represented by the Bragança (Bragança-Viseu and São Luís basins), Grajaú, Codó, and Itapecuru formations. In the studied sections, the Bragança (EGST-01 and VN-01 wells) and Codó (CI-01, PR-01, PE-01, and RL-01 wells) formations were recognized. The Bragança Formation consists of gray medium- to coarse-grained sandstones and conglomerates supported by conglomerates and greenish siltstone. This formation is interpreted as an alluvial fan deposit. The Codó Formation is composed of dark shales, anhydrite, and calcilutites, with sandstone intercalations. A lagoonal environment is assigned to these deposits. Marine incursions are indicated by the fossil content and the occurrence of evaporites.

Ceará Basin

The Ceará Basin is one of the equatorial margin basins formed during the breakup of the South American–African continent. The basin covers an area of 34,000 km^2 (Fig. 1). The Mundaú, Paracuru, and Ubarana formations represent the Cretaceous rocks of the basin. In the studied sections, the Mundaú (CES-17 well) and Paracuru (CES-27 and CES-44 wells) formations are recognized. The sediments of the Mundaú Formation were deposited in large

rift depressions and consist of conglomerates, sandstones, siltstones, and shales. The depositional environment of the formation is interpreted as continental (alluvial fans, rivers, and lakes). The Paracuru Formation consists of a continental to marine transitional sequence. Three main sets of facies are distinguished: a basal set composed of sandstones and shales, an intermediate layer consisting of carbonates (calcilutite, dolomite, and nodular limestones) and halite, and an upper layer of gray shales. The intermediate layer represents the Trairi Member assigned to the evaporitic phase, which is recognized only in the CES-44 well. The upper layer of the formation is recognized only in the CES-27 well. The three main facies of the formation represent sedimentation ranging from a deltaic environment to sabkha (Trairi Member) and epicontinental sea.

Potiguar Basin

The Potiguar Basin also belongs to the equatorial margin basins formed during the breakup of the South American–African continent. The basin covers an area of 60,000 km^2 (Fig. 1) at the eastern end of the equatorial margin, with 40% emerged. The Aptian succession is represented by the Pescada and Alagamar formations, but only the latter formation is recognized here. It is subdivided into two members (Upanema and Galinhos members), separated by a layer informally named the Camada Ponta do Tubarão (CPT). The basal Upanema Member consists of fine- to coarse-grained sandstones and shales, and the Galinhos Member is composed of dark gray shales. The two members and the CPT layer are well represented in the RNS-159 well. The environments are interpreted as ranging from fluvio-deltaic (Upanema Member), coastal lagoon/sabkha plain (CPT) to neritic (Galinhos Member).

Sergipe Basin

The Sergipe Basin in northeastern Brazil contains one of the most extensive Cretaceous marine carbonate successions among the central South Atlantic basins. The basin is 16–50 km wide, 170 km long, and covers an area of 6,000 km^2, and the offshore portion comprises an area of approximately 5,000 km^2 (Fig. 1). The upper Aptian is represented by the Muribeca and Riachuelo formations. The Muribeca Formation is composed of evaporites, clastics, and carbonate rocks. The formation is subdivided into three members, from base to top, the Carmópolis, Ibura, and Oiteirinhos members, of which the last two members are recognized in the studied sections (CL-47, GTP-17, and GTP-24 wells). The Ibura Member is represented by a succession of bituminous shales, evaporites (anhydrite, halite, tachyhydrite, carnallite, and sylvinite), and dolomitic limestones[78]. The Oiteirinhos Member consists of intercalations of gray to black bituminous shales, limestones, and siltstones.

The Riachuelo Formation is subdivided into three interdigitating members, viz. the Angico, Maruim, and Taquari members. The Angico Member is recognized in the CL-47 and GTP-17 wells. The Taquari Member is recognized only in the GTP-24 well. The Maruim Member has not been recognized. The Angico Member consists of very fine-grained sandstones to conglomerates interbedded with siltstones, shales, and rare thin beds of limestone[78], deposited nearshore by cyclical flows of shallow siliciclastic turbidities (delta fans). The Taquari Member was deposited during the open-marine phase and consists of rhythmically bedded, organic-rich, calcareous black shales and calcilutites.

Araripe Basin

The Araripe Basin is part of the Araripe–Potiguar Depression[79,80] and as such the largest inland basin in northeastern Brazil covering approximately 9,000 km^2 (Fig. 1)[79]. The basin is a globally well-known paleontological site studied since the end of the 19th century. The

Aptian is represented from the base to the top by the Barbalha, Crato, Ipubi, and Romualdo formations[81, 82]. In the studied sections, the last three formations are recognized. The sections 2-AP and PS-11[38] cover all three formations, whereas in the Sobradinho section[82] only the Romualdo Formation occurs. The Crato Formation consists of lacustrine laminated limestones with interbedded sandstones and shales. The Ipubi Formation is characterized by gypsum and anhydrite intercalated by dark shale beds representing sabkha plain and lagoonal deposits laid down under warm and semi-arid to arid conditions. The uppermost formation, the Romualdo Formation, is composed of shales and sandstones with thin limestone interbeds. The formation contains abundant fossils[79, 83-87] and is interpreted as a shallow-marine[82, 88] to outer-shelf environment[88].

The Espírito Santo Basin

The Espírito Santo Basin is located on the eastern margin of Brazil, in this study farthest from the humid belt of the equatorial region. The basin covers an area of approximately 218,000 km^2 (Fig. 1). The Aptian is represented by the Mariricu Formation and the base of the São Mateus and Regência formations, all recognized in the studied sections (PEI-03 and PEI-06 wells). The Itaúnas Member of the Mariricu Formation consists of carbonates, anhydrites, and halites and represent the evaporitic phase of the basin. The São Mateus Formation consists predominantly of sandstones deposited by alluvial and coastal fans in a marine environment. The Regência Formation consists of limestones (calcilutites and calcarenites) deposited on a shelf.

Samples

The palynological dataset comes from eight Brazilian sedimentary basins covering an area of approximately 425,000 km^2. A total of 555 core samples used in this study derive from 18

sections, of which sixteen wells drilled by the Brazilian oil company Petrobras, one well drilled by the Brazilian Geological Survey[38], and one outcrop described by[82] (Table S1). The slides of VN-1, EGST-1, RL-1, PE-1, CI-1, PR-1, CL-47, CES-17, CES-27, CES-44, RNS-159, 2-AP, PEI-03 and PEI-06 wells are kept at CENPES, Petrobras, Rio de Janeiro Brazil. The PS-11 slides are kept in the laboratory LABMICRO of the Department of Geology of the Federal University of Rio de Janeiro, Rio de Janeiro. The slides of the Sobradinho section are housed in the São Paulo State University (UNESP) collection, IGCE-UNESPetro, Rio Claro, SP. The GTP-17 and GTP-24 slides have been used in previous studies, although analyzed with other methods[6, 7]. The slides were kept at the Museu Nacional in Rio de Janeiro and unfortunately destroyed in a fire on September 2, 2018.

Composite sections

All 18 sections were analyzed individually for palynology. However, composite sections were constructed by sedimentary basins (Table S1), based on the stratigraphically evident chronological distribution of paleoclimatic phases (pre-evaporitic, evaporitic, and post-evaporitic) for each section studied, i.e., 2-AP (17 samples), PS-11 (38 samples), and Sobradinho (38 samples) for the Araripe Basin (a total of 93 samples); 2-EGST (8 samples), 2-VN (4 samples), CI-01 (13 samples), PE-01 (4 samples), PR-01 (4 samples) and RL-01 (7 samples) for the Bragança-Viseu, São Luís and Parnaíba basins (40 samples); CES-17 (8 samples), CES-27 (7 samples) and CES-44 (18 samples) for the Ceará Basin (33 samples); PEI-03 (11 samples) and PEI-06 (14 samples) for the Espírito Santo Basin (25 samples); RNS-159 (41 samples) for the Potiguar Basin (41 samples) and CL-47 (68 samples), GTP-17 (102 samples) and GTP-24 (153 samples) for the Sergipe Basin (320 samples).

Supplementary text

Palynological dataset

A total of 139 genera were recorded in the 555 samples, distributed into five plant groups: bryophytes (five genera), ferns (58 genera), lycophytes (18 genera), pteridosperms (one genus), gymnosperms (27 genera), and angiosperms (30 genera) (Table S2). Of these, 19 indeterminate morphotypes were found in ferns and 10 in angiosperms. Of the 139 genera recognized, the botanical affinities at the family level reached 100% in bryophytes, 56.9% in ferns, 100% in lycophytes, 92.6% in gymnosperms, and 40.0% in angiosperms, totaling 67.6% (Table S2).

Bioclimatic groups

Hygrophytes

Hygrophyte plants depend on water to reproduce and are therefore generally associated with moist conditions and rarely reported from arid environments. The hygrophyte group was the most diverse of all the flora recorded in the sections studied. The group is composed of 51 genera of ferns and 13 genera of lycophytes. Notably, *Cicatricosisporites* was the most abundant genus in the studied sections, well representing hygrophyte plants. The spores of *Cicatricosisporites* are assigned to the family Anemiaceae (*78*), containing only one genus, *Anemia*. The genus occurs mainly in the coastal regions of tropical and subtropical America[90, 91]. *Cicatricosisporites*, as indicator of humid climatic conditions (tropical climates), is extensively documented in the literature[6, 7, 91-94]. Among the lycophytes, the genus *Retitriletes*, assigned to the family Lycopodiaceae, is similar to the modern genus *Lycopodium*, which occurs in humid environments worldwide.

Hydrophytes

Hydrophytes represent aquatic plants that live with a portion of their vegetative parts permanently immersed in water. These plants (herbaceous and rhizomatous) present themselves in three main ways: 1) floating - their short and simple roots do not anchor to the bottom, and the entire plant remains floating on the surface, with a high rate of transpiration (e.g., *Salvinia*, *Azolla*). They are generally restricted to sheltered habitats and weak current watercourses[95]; 2) semi-aquatic parts of the plant remain out of the water (leaves and flowers), but at least the roots are attached to the bottom. They usually have short stems or underground rhizomes attached to the substrate and long leaves or petioles that keep their leaves out of the water (e.g., lotus, water lilies, *Typha*). In general, they are found at shallow depths, from 0.5 m to 3 m[96]; 3) submerged - its roots can be fixed to the bottom or free, and the entire body of the plant remains under water. They possess very fine and delicate structures, usually with small leaves, without any type of support fabric (e.g., *Cabomba*). They can be found at all depths within the photic zone, with little turbulence and turbidity for photosynthetic activity.

Among spore-producing aquatic plants, the genus *Crybelosporites* is the most abundant. *Collarisporites*, *Gabonisporis*, and *Paludites* of the family Marsileaceae (Table S2) are common in the Lower Cretaceous. This family includes aquatic plants with roots attached to the substrate[97-99]. *Crybelosporites pannuceus* were produced by the marsile plant *Mendozaphyllum loncochense*, as described by[89] for the Campanian–Maastrichtian Loncoche Formation of the Neuquén Basin, Argentina. The recent family Marsileaceae is a rhizomatous plant rooted in shallow-water substrates, with parts that extend to the water surface[100]. They occur in shallow waters, often in small temporary ponds. In the section studied by[99], the spores of *C. pannuceus* occur abundantly in pelitic sediments interpreted as deposited in fluvial to deltaic environments, with lacustrine episodes. According to the refers.[97-101], this seems to be ideal for the full development of aquatic ferns related to the Marsileaceae

(perisporate spores) and Salviniaceae (spore masses) families. This group also includes the lycophyte Selaginellaceae (*Densoisporites*, *Perotrilites*) and the Isoetaceae (*Echitriletes*) (Table S2).

Bryophytes are poikilohydric plants that do not control water loss and are intolerant to desiccation. They live only in environments with permanently shallow water, but decay when the environment dries[100]. In the studied sections the bryophytes, represented mainly by the genus *Aequitriradites* (Table S2), are quantitatively insignificant; they occur associated with perisporate Marsileaceae spores, thus reinforcing the ecological behavior of aquatic habits.

Tropical lowland flora

The tropical lowland flora in this study is attributed to 37 families related to more humid conditions in lowland areas. All 30 angiosperm genera and morphotypes are included in this flora, in addition to seven gymnosperm genera. Botanical affinities allowed us to include these genera in the group. The most abundant genus is *Afropollis,* which is attributed to angiosperms, although still under debate. The parent plants of *Afropollis* thrived better in wetter than dry tropical conditions, being able to colonize environments with intermittent sedimentation, such as a floodplain[7, 29, 32, 102].

The angiosperm group is characterized by low abundance and moderate diversity. The most abundant genus is *Afropollis,* although not exceeding 1.0% of all palynomorphs in the studied sections. Most angiosperm genera are represented by only a single grain.

Another important family in tropical lowland areas is the Euphorbiaceae (*Psiladicolpites*), cosmopolitan dicots, which preferentially inhabited tropical regions. The largest dispersion centers are found in the Americas and Africa and are well represented in the Brazilian flora[54]. The Euphorbiaceae are a large family with very varying habits, including herbs, shrubs, and trees. The Arecaceae (palms), with three genera, was the most diverse family (Table S2). The

current distribution of the family closely coincides with that of tropical and subtropical climates. Palms have been widely used as paleo-indicators of warm and humid climates[103-106]. Gymnosperms are represented only by the family Cycadaceae (*Cycadopites*), with a distribution similar to palms, i.e., tropical regions.

Upland flora

Families assigned to thermophilic, large conifers, such as the Araucariaceae (*Araucariacites*) and Podocarpaceae (*Caliallasporites*), formed forests in the highlands, dominating vegetation between 900 and 1,800 m altitude. A total of 19 genera (five ferns and 14 gymnosperms) are included in the upland flora (Table S2). *Araucariacites* is the second most abundant genus, with 13,354 occurrences. Together with *Araucariacites*, a number of other genera (e.g., *Balmeiopsis* and *Cingulatipollenites*) are assigned to the Araucariaceae. Today, the family has a dominantly austral distribution, whereas in the Mesozoic, the family was distributed in both the northern and southern hemispheres[107]. Most modern representatives of the Araucariaceae live in areas with a subtropical or temperate mesothermic climate. However, in New Caledonia the genus *Araucaria* grows down to sea level. The family is typical of highlands and related to a tropical group whose pollen species are found in lowland deposits of Early Cretaceous age[32]. Araucariaceae when associated with Podocarpaceae suggest a tropical or subtropical mountain climate[108].

In the transitional area, the altitude range between 200 and 600 m was probably inhabited by representatives of the family Cupressaceae, mainly represented by *Inaperturopollenites*. Today, this family does not occur naturally in Brazil; however, it was relatively abundant in the Cretaceous.

Xerophytes

Xerophytes represent the most abundant group of plants in the studied sections. Unlike hygrophytes and hydrophytes, the group is adapted to xeric or water-stressed environments and therefore associated with arid climates. The xerophytes are dominated by warmth-loving conifers of the family Cheirolepidiaceae and *Classopollis* pollen, attesting to arid conditions. *Classopollis* dominates mainly in lagoonal and marine nearshore environments, often associated with evaporites[30-34, 109]. As described in[35], the unusual leaf morphology of the family, which is reminiscent of angiosperm halophytes such as *Salicornia*[35, 110], further suggests an arid and hot climate. According to refer.[111], the Cheirolepidiaceae inhabited open and sunny lowland areas, relatively close to water bodies (lake or coastal marine), such as alluvial and delta and coastal areas (with well-drained and dry soils), and are not representative of swampy or humid soils[31, 111]. The xerophyte group herein is composed of eight genera, dominated by *Classopollis* (Cheirolepidiaceae). This genus is conspicuously the most abundant genus identified in this study, with 45,867 occurrences. Besides *Classopollis*, the xerophytes are represented by ephedroid pollen grains of the families Ephedraceae and Gnetaceae (*Equisetosporites*, *Gnetaceaepollenites*, and *Steevesipollenites*) (Table S2). Today, the family Ephedraceae contains only one genus, *Ephedra*, which occurs in the arid regions of America, Asia, Europe, and North Africa.

Stratigraphic distribution of the bioclimatic groups

The Bragança-Viseu, São Luís, and Parnaíba basins

The composite section of the Bragança-Viseu, São Luís, and Parnaíba basins consists of 40 samples, with 24 samples from the pre-evaporitic phase, eight from the evaporitic phase, and eight from the post-evaporitic phase (Table S1). In general, the composite section highlights

the bioclimatic groups of hygrophytes (18.8%) and tropical lowland flora (Table S9). The diversity and Fs/X ratio curves showed strong synchrony (Fig. S1), indicating a relation between diversity and humidity. No marine elements were recorded in these sections.

The pre-evaporitic phase: This phase is characterized by a higher abundance of xerophytes, hygrophytes, and upland flora. However, the curves of these groups show strong oscillations. The dendrogram (Fig. S1) reveals two intervals in this phase, with significant values of xerophytes at the base but with a decreasing trend towards the top. The interval above the xerophyte curve shows an upward trend. The diversity and Fs/X ratio curves show synchrony between them, but with a decreasing trend towards the top.

The evaporitic phase: This phase is related to the gypsum layers of the Codó Formation and marked by the highest average of the xerophytic in the composite section (Table S9). Also noteworthy is the high average of the tropical lowland flora bioclimatic group, driven by the genus *Afropollis*. Unexpectedly, the mean diversity is high, but in this case it is not matched by the mean Fs/X ratio, which shows the lowest in the section. The high diversity in more arid conditions is probably related to the occurrence of species of *Classopollis* and *Equisetosporites*.

The post-evaporitic phase: In this phase, the lowest mean of xerophyte species (47.4%) and the highest mean of upland flora were recorded. The dendrogram reveals the main break between this phase and the evaporitic phase (Fig. S1). In general, this reflects an inversion of abundance between groups related to humidity (hygrophytes, hydrophytes, tropical flora, upland flora) and groups related to drier conditions (xerophytes) (Fig. S1).

The Ceará Basin

The composite section of the Ceará Basin consists of 33 samples, with eight samples from the pre-evaporitic phase, 18 from the evaporitic phase, and seven from the post-evaporitic

phase (Table S1). In general, the composite section highlights the hygrophytes and hydrophytes bioclimatic groups (Table S10). The diversity and Fs/X ratio curves show strong synchrony, indicating a relation between diversity and humidity. Marine elements are present in the post-evaporitic phase.

The pre-evaporitic phase: This phase is characterized by the highest average of hydrophytes in the studied basins, with the highest peaks related to a significant decrease in xerophytes (Fig. S2), accompanied by the highest values for the Fs/X ratio. In this phase, the highest diversity indices were recorded, reaching an average of H’= 2.5.

The evaporitic phase: This phase also recorded the highest xerophytic values (66.0%) (Table S10), similar to the sections of the Bragança-Viseu, São Luís, and Parnaíba basins. The tropical lowland flora bioclimatic group also presents an average slightly higher than the general average. These data confirm the relationship between *Afropollis* and warmer conditions. All other groups related to more humid conditions had values below the average. The diversity and Fs/X ratio indices also show values below the mean.

The post-evaporitic phase: In this phase, the highest averages of bioclimatic groups related to increased humidity (hygrophytes, upland flora) were recorded, accompanied by one of the lowest averages of the xerophyte groups (35.3%) (Table S10). The dendrogram for this section also reveals that the main break occurs between this phase and the previous one (Fig. S2), generally reflecting an inversion of abundance between the groups related to moisture (hygrophytes, hydrophytes, tropical flora, upland flora) and drier conditions (xerophytes) (Fig. S2). An abrupt decrease in the xerophyte bioclimatic group was recorded. The diversity and Fs/X ratio indices show an upward trend toward the top of the section (Fig. S2).

The Potiguar Basin

The composite section of the Potiguar Basin consists of 41 samples, with 29 samples from the pre-evaporitic phase, six from the evaporitic phase, and six from the post-evaporitic phase (Table S1). In general, the hygrophyte bioclimatic group stands out in the composite profile of this basin (Table S11), especially at the base of the section (Fig. S3). The diversity and Fs/X ratio curves show strong synchrony, indicating a relationship between diversity and moisture. Marine elements were recorded only in the post-evaporite phase. (Fig. S3).

The pre-evaporitic phase: In the basin section, this phase occurs within the Upanema Member and is characterized by high averages of the hygrophyte and upland flora bioclimatic groups (Table S11). However, these groups show a downward trend toward the top of this phase. An important feature is the lowest xerophyte value recorded for all the studied basins. The diversity and Fs/X ratio initially also show an upward trend, followed by a downward trend toward the top of the phase (Fig. S3). By contrast, in this phase the xerophytes show low values, mainly at the base of the phase, but with a progressive tendency to increase. No marine elements were observed during this stage.

The evaporitic phase: In this phase, only the xerophyte bioclimatic group show an average higher than the general average of the section. This group show an increasing trend within the phase, reaching an apex within the Camada Ponta do Tubarão (Fig. S3). However, even in the evaporitic phase, it shows a downward trend toward the top of the phase. The diversity and Fs/X ratio indices also show values below the mean.

The post-evaporitic phase: In this phase, only the upland flora bioclimatic group shows an average greater than the general average of the section. The largest break in the dendrogram occurs within this phase, in the Galinhos Member. However, the break does not coincide with the limit between this phase and the evaporitic phase, as in the other sections (Fig. S3), but with a sudden drop in xerophytes to the detriment of the increase in upland flora. The

diversity and Fs/X ratio indices show contrasting trends, with the first being above the mean and the second below the mean.

The Sergipe Basin

The composite section of the Sergipe Basin consists of 323 samples, with 92 from the evaporitic phase and 231 from the post-evaporitic phase (Table S1). In general, the composite section of the basin highlights the xerophyte bioclimatic group (Table S12), especially at the base, and the hygrophyte and upland flora groups at the top of the section. The diversity and Fs/X ratio curves show strong synchrony, indicating a relationship between diversity and moisture. Marine elements are recorded in all phases (Fig. S5) and particularly abundant and diversified in the post-evaporitic phase.

The evaporitic phase: In this phase, represented by the Ibura Member, the xerophyte bioclimatic group registers one of the highest averages of all sections (84.4%) (Table S12). However, as shown in Fig. S4, there was a gradual increase in upland flora. The diversity indices and Fs/X ratio show values below the mean, but the curves also indicate an increase toward the top of the phase.

The post-evaporitic phase: In this phase, although xerophytes remain at high values, as indicated by the curve (Fig. S4), even in the Oiteirinhos Member, there is a sudden drop in this group accompanied by a marked increase in upland flora (e.g., *Araucariacites*). The most extensive break in the dendrogram occurs within this phase and reflects the progressive decrease of xerophytes accompanied by the progressive increase of hygrophytes and the upland flora bioclimatic group. This trend was observed in the Angico and Taquari members (Fig. S4). The xerophyte drop intervals are accompanied by an increase in the diversity indices and the Fs/X ratio, which register values above the average.

The Araripe Basin

The composite section of the Araripe Basin consists of 93 samples, with 27 samples from the pre-evaporitic phase, 12 from the evaporitic phase, and 54 from the post-evaporitic phase (Table S1). In general, the composite profile of the basin shows prominent values of the hygrophyte bioclimatic group (Table S13). The diversity and Fs/X ratio curves show strong synchrony, indicating a relationship between diversity and moisture. Marine elements occur in all three phases.

The pre-evaporitic phase: This phase, recorded in the Crato Formation, is characterized by one of the highest averages of hygrophytes in the studied basins, with the highest peaks related to a significant decrease in xerophytes (Fig. S5), accompanied by the highest values of the Fs/X ratio. Average diversity indices were below the overall average. In this phase, the dendrogram (Fig. S5) reveals three intervals, evidenced by the prominent oscillation of the xerophyte bioclimatic group. In the basal and upper intervals, the highest values of xerophytes are recorded. The intermediate interval is marked by a significant increase in the upland flora. Marine elements (dinoflagellate cysts) were recorded, especially in the 2AP well.

The evaporitic phase: This phase is marked by the highest mean of the xerophyte bioclimatic group corresponding to part of the Ipubi Formation (Fig. S5). The diversity and Fs/X curves show a decreasing trend during this phase. Dinoflagellate cysts are reported in the PS-11 section.

The post-evaporitic phase: This phase corresponds to the Romualdo Formation, where high average of the xerophyte group is still recorded, although with a sharp drop, almost reaching zero (Fig. S5). In this interval, the dendrogram reveals the main break, related to the inversion of abundance between xerophytes and upland flora (Fig. S5). Subsequently, the group recovers and again shows a tendency to decrease toward the top of the section, although progressively. The hydrophytes show an average above the overall average, indicating wet

conditions. The upland flora bioclimatic group also shows an average higher than the general average. The upland flora was accompanied by one of the lowest xerophyte averages (35.3%) (Table S13). The diversity and Fs/X ratio indices show an upward trend toward the top of the section (Fig. S5) and are prominent where xerophytes show lower values. In this phase, significant values of marine elements are recorded. In section PS-11, a total of 306 specimens of *Subtilisphaera* were recorded at a depth of 4.4 m, with high values (above 200 specimens) also recorded in samples 10.0, 12.4, and 15.0 m, which characterize a bloom commonly observed in the upper Aptian[112].

The Espírito Santo Basin

The composite section of the Espírito Santo Basin consists of 25 samples, of which 11 from the evaporitic phase and 14 from the post-evaporitic phase (Table S1). In general, this section is strongly dominated by the xerophyte bioclimatic group. The highest averages of xerophytes among all the basins studied were recorded in the Araripe Basin. (Table S14), and the curves of the bioclimatic groups do not show strong oscillations. The diversity and Fs/X ratio curves also show strong synchrony. Marine elements were recorded in both phases. (Fig. S6).

The evaporitic phase: In this phase, within the Itaúnas Member, the xerophyte bioclimatic group records one of the highest average abundances of all sections studied (84.9%) (Table S14). A significant peak of hygrophytes was recorded slightly below the limit between this phase and the post-evaporitic phase, as confirmed by the break in the dendrogram (Fig. S6). The diversity indices and Fs/X ratio show the same values for all sections. Marine elements are present but with below-average values.

The post-evaporitic phase: In this phase, xerophytes dominate; however, as shown in Fig. S6, there is a slight oscillation at the base, with a slight increase in the hygrophyte and tropical

lowland flora groups. In this inflection of the curves, the main break of the dendrogram was recorded (Fig. S6). After the breakup, xerophytes again show a trend of progressive increase, but there is a new oscillation in this group at the top of the section, accompanied by a slight increase in the hygrophyte, tropical lowland flora, and hydrophyte groups, which present values above the section average. The diversity indices and Fs/X ratio show the same low values as in the previous phase. Marine elements are present but with above-average values.

References

77. H. K. Chang, R. O. Kowsmann, A. M. F. Figueiredo, New concepts on the development of east Brazilian marginal basins. *Episodes* **11**, 194–202 (1988). 38

78. E. A. M. Koutsoukos, *Mid- to Late Cretaceous Microbiostratigraphy, Palaeo-ecology and Palaeogeography of the Sergipe Basin, Northeastern Brazil*. PhD thesis, Council for National Academic Awards, Polytechnic South West, Plymouth, UK (1989).

79. M. L. Assine, Bacia do Araripe. *B. Geol. Petrobras* **15**, 371–389 (2008; for 2007).

80. J. M. Mabessone, I. M. Tinoco, Paleoecology of the Aptian Santana Formation (northeastern Brazil). *Palaeogeogr. Palaeoclimatol. Palaeoecol.* **14**, 97–118 (1973).

81. V. H. Neumann, L. Cabrera, J. M. Mabesoone, L. M. M. Valença, A. L. Silva, Ambiente sedimentar e facies da seqüência lacustre aptiana-albiana da Bacia do Araripe, NE do Brasil. *Boletim do 6° Simpósio sobre o Cretáceo do Brasil, São Pedro, SP*, pp. 37–41 (2002).

82. M. Arai, M. L. Assine, Chronostratigraphic constraints and paleoenvironmental interpretation of the Romualdo Formation (Santana Group, Araripe Basin, northeastern Brazil) based on palynology. *Cretaceous Res.* **116**, 104610 (2020).

83. F. C. Ponte, C. J. Appi, Proposta de revisão da coluna litoestratigráfica da Bacia do Araripe. *Anais do 36 Congresso Brasileiro de Geologia, Natal, RN, Vol. 1*, pp. 211–226 (1990).

84. F. C. Ponte, F. C. Ponte Filho, "Estrutura geológica e evolução tectônica da Bacia do Araripe" (Tech. Rep. DNPM, Recife, PE, 1996).

85. V. H. Neumann, L. Cabrera, Una nueva propuesta estratigráfica para la tectonosecuencia post-rifte de la Cuenca de Araripe, Noreste de Brasil. *Boletim do 5° Simpósio sobre o Cretáceo do Brasil, Serra Negra, SP,* pp. 279–285 (1999).

86. J. C. Coimbra, M. Arai, A. L. Carreño, Biostratigraphy of Lower Cretaceous microfossils from the Araripe Basin, northeastern Brazil. *Geobios* **35**, 687–698 (2002).

87. M. A. Custódio, F. Quaglio, L. V. Warren, M. G. Simões, F. T. Fürsich, J. A. J. Perinotto, M. L. Assine, The transgressive-regressive cycle of the Romualdo Formation (Araripe Basin): sedimentary archive of the Early Cretaceous marine ingression in the interior of northeast Brazil. *Sediment. Geol.* **359**, 1–15 (2017).

88. R. M. Melo, J. Guzmán, D. Almeida-Lima, E. K. Piovesan, V. H. M. L. Neumann, A. J. Sousa, New marine data and age accuracy of the Romualdo Formation, Araripe Basin, Brazil. *Sci. Rep.* **10**, 15779 (2020).

89. A. R. Smith, K. M. Pryer, E. Schuettpelz, P. Korall, H. Schneider, P. G. Wolf, A classification of extant ferns. *Taxon* **55**, 705–731 (2006).

90. R. M. Tryon, A. F. Tryon, *Ferns and Allied Plants with Special Reference to Tropical America*. (Springer-Verlag, New York, 1982).

91. P. L. Narváez, N. Mego, M. B. Prámparo, Cretaceous cicatricose spores from north and central western Argentina: taxonomic and biostratigraphical discussion. *Palynology* **37**, 202–217 (2013).

92. L. C. Ruiz, M. E. Quatrocchio, *Srivastavapollenites exoticus* nov. gen. et sp. de la Formación Pedro Luro (Paleoceno), Cuenca del Colorado, Argentina. *Ameghiniana* **30**, 311–315 (1993).

93. M. B. Prámparo, W. Volkheimer, Nuevos hallazgos de palinomorfos en la Formación La Amarga, Miembro Bañados de Caichigüe, Cuenca Neuquina sudoriental, Argentina. *Ameghiniana* **39**, 395–404 (2002).

94. S. G. Duarte, F. J. Silva, M. Arai, L. S. Sylvestre, M. D. Wanderley, N. Jha, H. Joshie, E. Masure, H. El Atfy. Paleoclimatic, paleoenvironmental and paleoecological implications of the Family Anemiaceae: A palynological investigation from variable spatial and temporal strata. *Rev. Palaeobot. Palyno.* **285**, 104316 (2020).

95. R.G. Wetzel, *Limnologia*. (Fundação Calouste Gulbenkian, Lisbon ed. 2., 1993).

96. F. A. Esteves, *Fundamentos de Limnologia* (Interciência/FINEP. Rio de Janeiro, ed. 2, 1998).

97. A. M. Baldoni, Palynology of the lower Lefipa Formation (Upper Cretaceous) of Barranca de Los Perros, Chubut Province, Argentina. Part I. Cryptogam spores and gymnosperm pollen. *Palynology* **16**,117–136 (1992).

98. M. E. Collinson, S. Y. Smith, J. H. A. Van Konijnenburg-Van Cittert, D. J. Batten, J. van der Burgh, J. Barke, F. Marone, New observations and synthesis of Paleogene heterosporous water ferns. *Int. J. Plant Sci.* **174**, 350–363 (2013).

99. N. R. Cúneo, M. A. Gandolfo, M. C. Zamaloa, E. Hermsen, Late Cretaceous Aquatic Plant World in Patagonia, Argentina. *PLOS ONE* **9**, e104749 (2014).

100. G. G. Puebla, M. B. Pramparo, M. A. Gandolfo, Aquatic ferns from the Upper Cretaceous Loncoche Formation, Mendoza, central-western Argentina. *Plant Syst. Evol.* **301**, 577–588 (2015).

101. G. G. Puebla, N. Mego, M. B. Prámparo, Asociación de briófitas de la Formación La Cantera, Aptiano tardio, Cuenca de San Luis, Argentina. *Ameghiniana* **49**, 217–22 (2012).

102. N. N. Ferreira, E. P. Ferreira, R. R. C. Ramos, I. S. Carvalho, Terrestrial and marine palynomorphs from deposits of the pull-apart rift of West Gondwana (Parnaíba Basin, northern Brazil): Biostratigraphy and relation to tectonic events. *J. S. Am. Earth Sci.* **101**, 102612 (2020).

103. D. R. Greenwood, S. L. Wing, Eocene continental climates and latitudinal temperature gradients. *Geology* **23**, 1044–1048 (1995).

104. R. J. Morley, *Origin and Evolution of Tropical Rain Forests*. (John Wiley & Sons, Chichester, UK, 2000).

105. R. J. Morley, Interplate dispersal paths for megathermal angiosperms. Perspectives in Plant Ecology, *Evol. Syst.* **6**, 5–20 (2003).

106. G. R. Walther, E. S. Gritti, S. Berger, T. Hickler, Z. Y. Tang, M. T. Sykes, Palms tracking climate change. *Global Ecol. Biogeogr.* **16**, 801–809 (2007).

107. T. L. Dutra, A. Stranz, T. P. Wilberger, “Araucariaceae: phytohistory of a family” in *Brief History of the Gymnosperms: Classification, Biodiversity, Phytogeography and Ecology*, J. M. Anderson, H. M. Anderson, C. J. Cleal, Eds., National Biodiversity Institute, Pretoria, pp. 56–59 (2007).

108. Y. Reyre, Peut-on estimer l'évolution des climats Jurassiques et Crétacés d'apres la palynologie? Mém. Mus. natl. hist. nat., Sér. B Bot. **XXVII**, 247–260 (1980).

109. V. A. Vakhrameev, Range and paleoecology of Mesozoic conifers. The Cheirolepidiaceae. *Paleontol. J.* **41**, 11–25 (1970).

110. K. L. Alvin, Cheirolepidiaceae: biology, structure and paleoecology. *Rev. Palaeobot. Palynol.* **37**, 71–98 (1982).

111. V. A. Vakhrameyev, *Classopollis* pollen as an indicator of Jurassic and Cretaceous climate. *Int. Geol. Rev.* **24**, 1190–1196 (1982). (Translated from В.А. Вахрамеев, Пыльца *Classopollis* как индикатор климата юры и мела. *Советская Геология* **8**, 48–56 (1980).

112. M. Arai, J. Botelho Neto, C. C. Lana, E. Pedrão, Cretaceous dinoflagellate provincialism in Brazilian marginal basins, *Cretaceous Res.* **21**, 351–366 (2000).

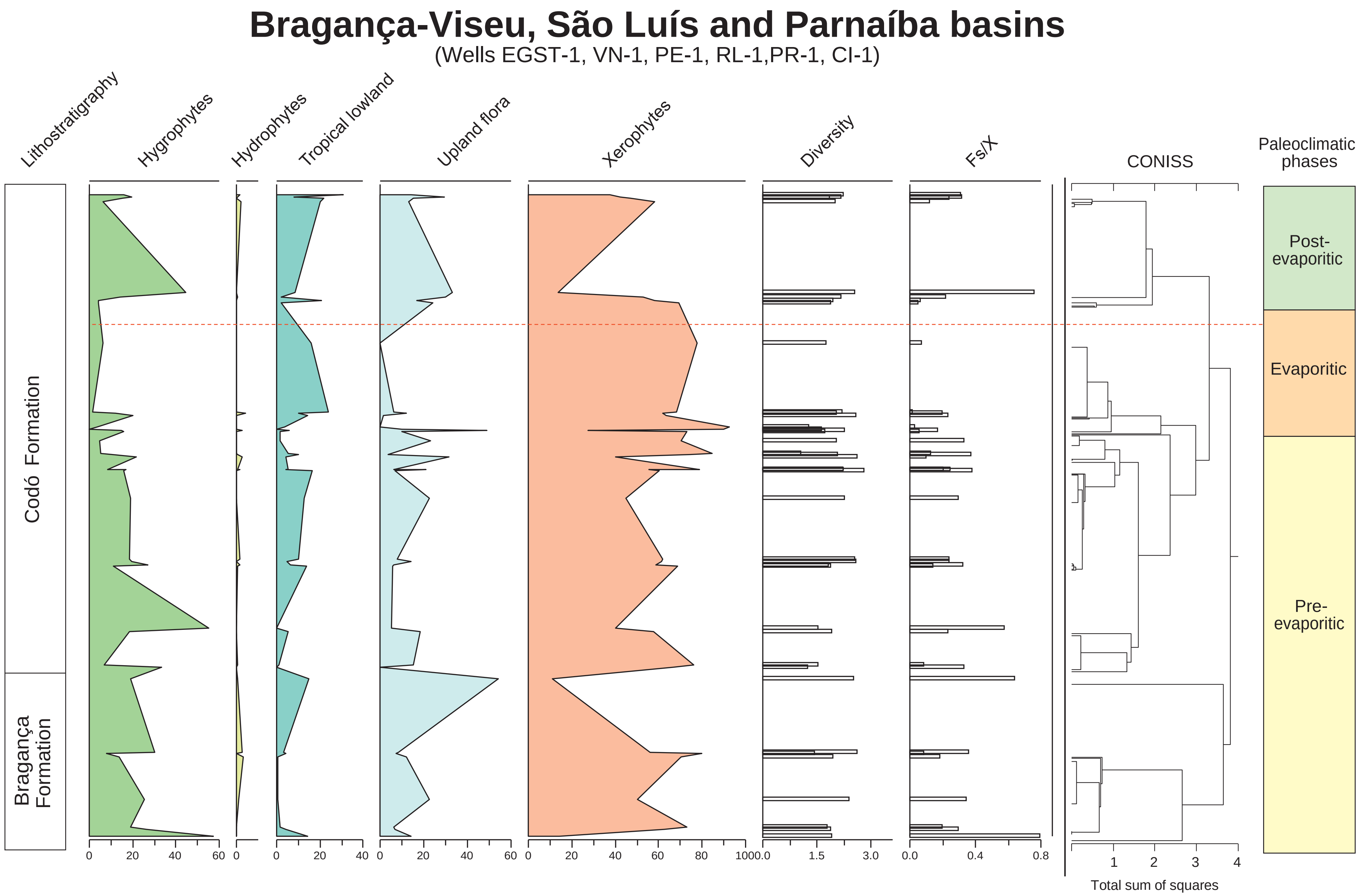

Fig. S1.
Changes in composition and diversity of the late Aptian palynoflora of the Bragança Viseu, São Luís and Parnaíba basins. Changes in bioclimatic groups, diversity, Fs/X against the paleoclimatic phases. Agglomerative, hierarchical clustering and stratigraphically constrained dendrogram (CONISS) showing the main break (dashed red line).

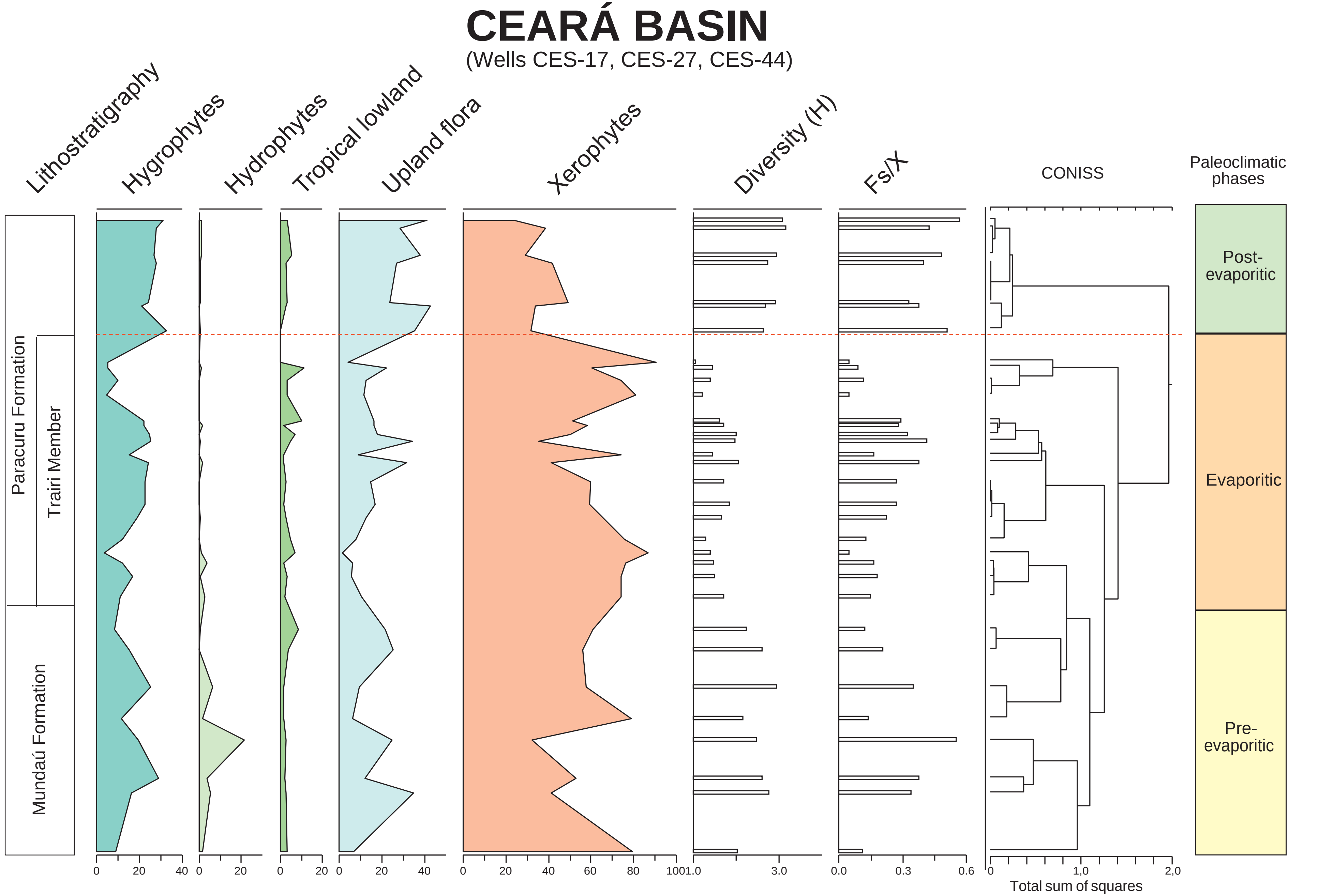

Fig. S2.

Changes in composition and diversity of the late Aptian palynoflora of the Ceará basins. Changes in bioclimatic groups, diversity, Fs/X against the paleoclimatic phases. Agglomerative, hierarchical clustering and stratigraphically constrained dendrogram (CONISS) showing the main break (dashed red line).

POTIGUAR BASIN

(Well RNS-159)

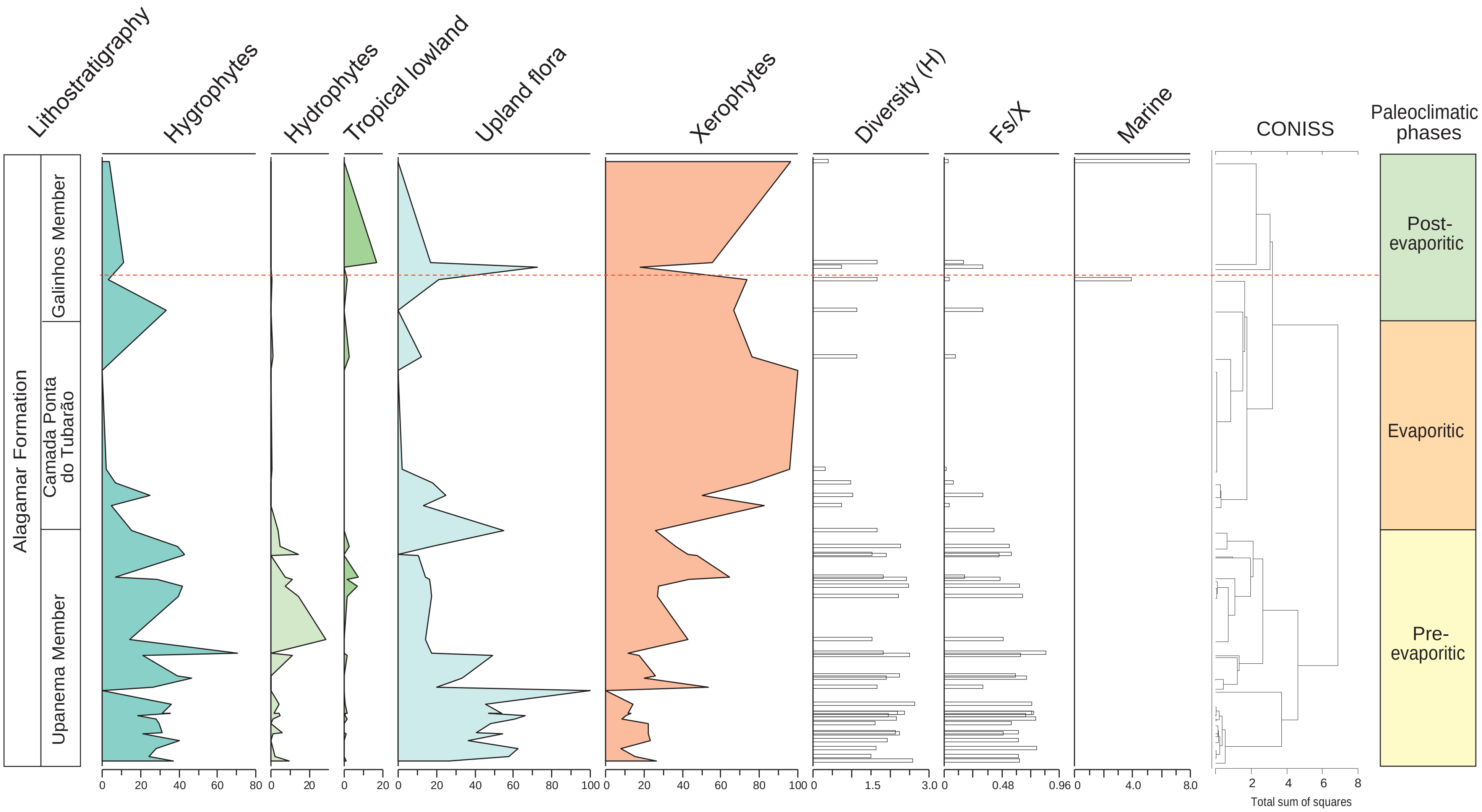

Fig. S3.
Changes in composition and diversity of the late Aptian palynoflora of the Potiguar basins. Changes in bioclimatic groups, diversity, Fs/X against the paleoclimatic phases. Agglomerative, hierarchical clustering and stratigraphically constrained dendrogram (CONISS) showing the main break (dashed red line).

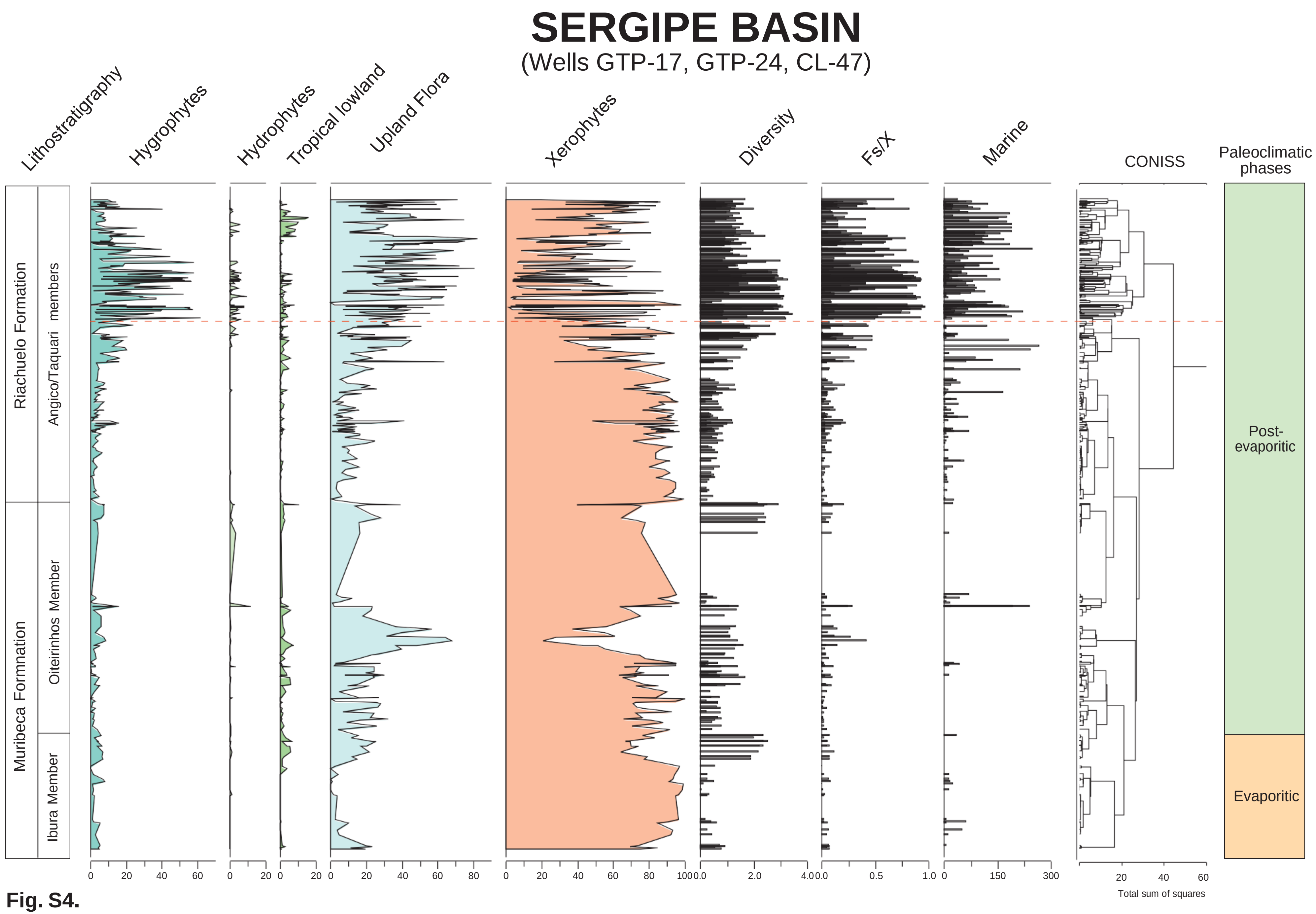

Fig. S4.
Changes in composition and diversity of the late Aptian palynoflora of the Sergipe basins. Changes in bioclimatic groups, diversity, Fs/X against the paleoclimatic phases. Agglomerative, hierarchical clustering and stratigraphically constrained dendrogram (CONISS) showing the main break (dashed red line).

ARARIPE BASIN

(Wells 2AP, PS-11, and section Sobradinho)

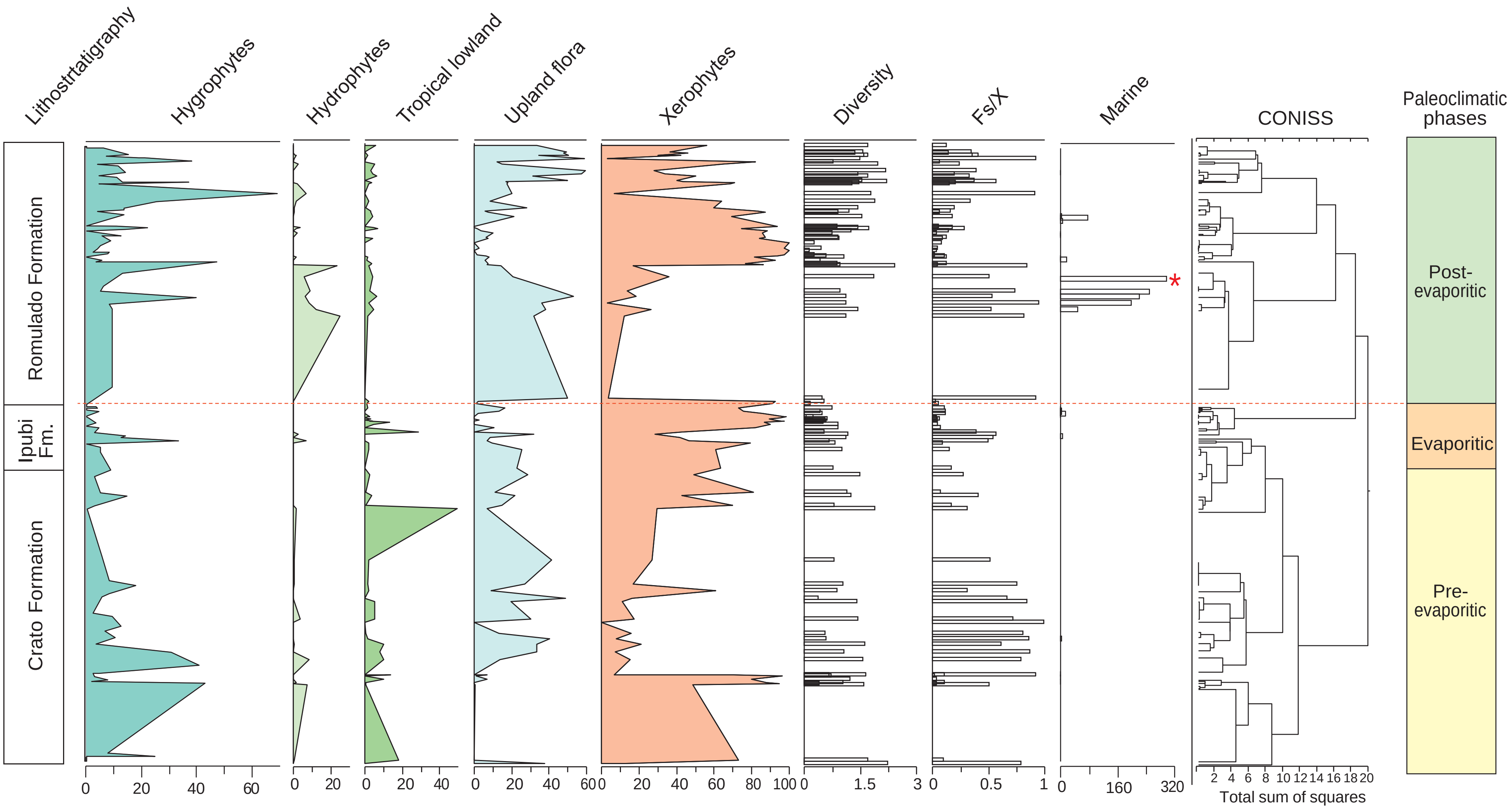

Fig. S5.
Changes in composition and diversity of the late Aptian palynoflora of the Araripe basins. Changes in bioclimatic groups, diversity, Fs/X against the paleoclimatic phases. Agglomerative, hierarchical clustering and stratigraphically constrained dendrogram (CONISS) showing the main break (dashed red line).

ESPÍRITO SANTO BASIN

(Wells PEI-3 and PEI-6)

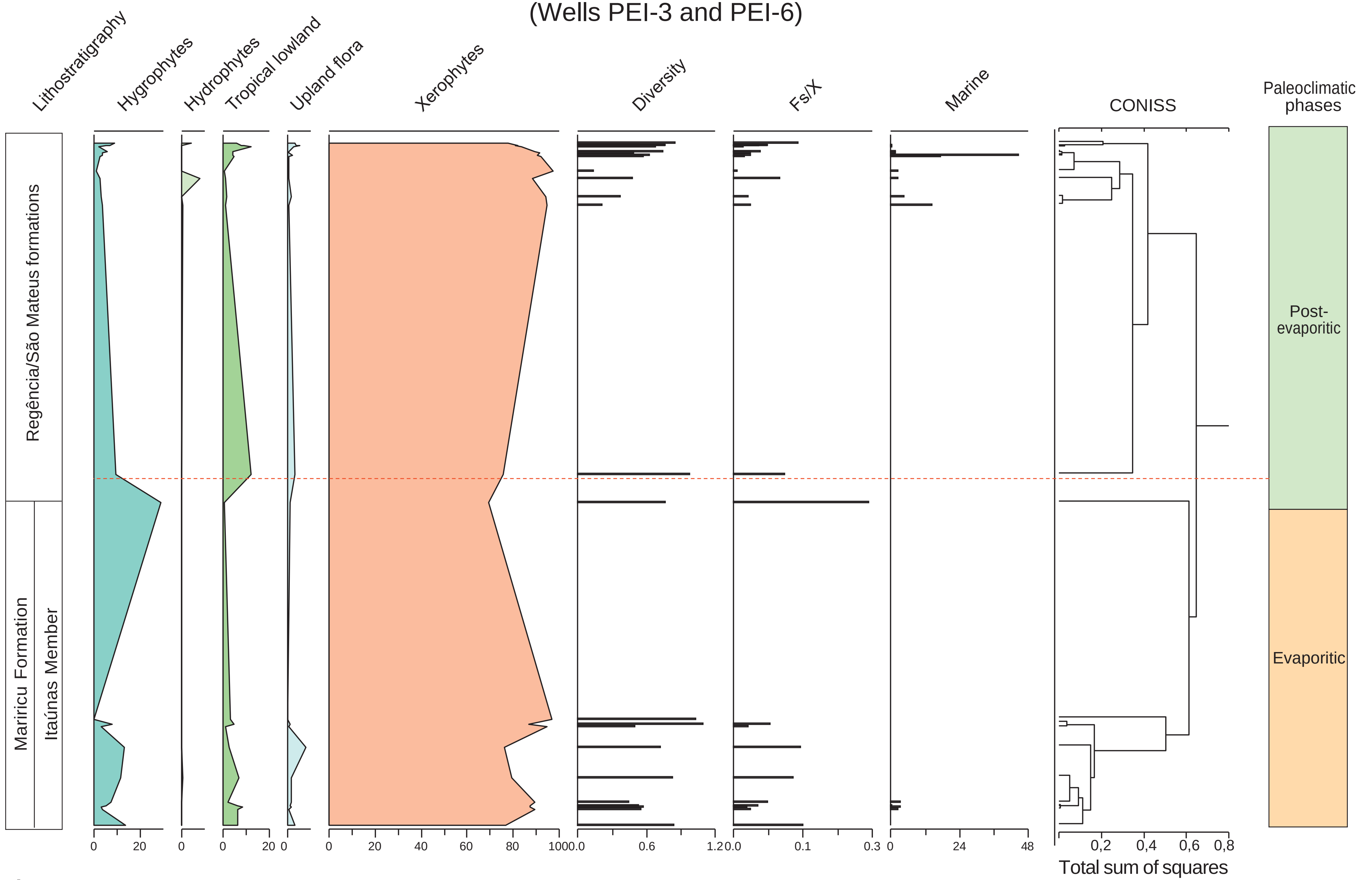

Fig. S6.
Changes in composition and diversity of the late Aptian palynoflora of the Espírito Santo basins. Changes in bioclimatic groups, diversity, Fs/X against the paleoclimatic phases. Agglomerative, hierarchical clustering and stratigraphically constrained dendrogram $(CONISS_2)_8$ showing the main break (dashed red line).

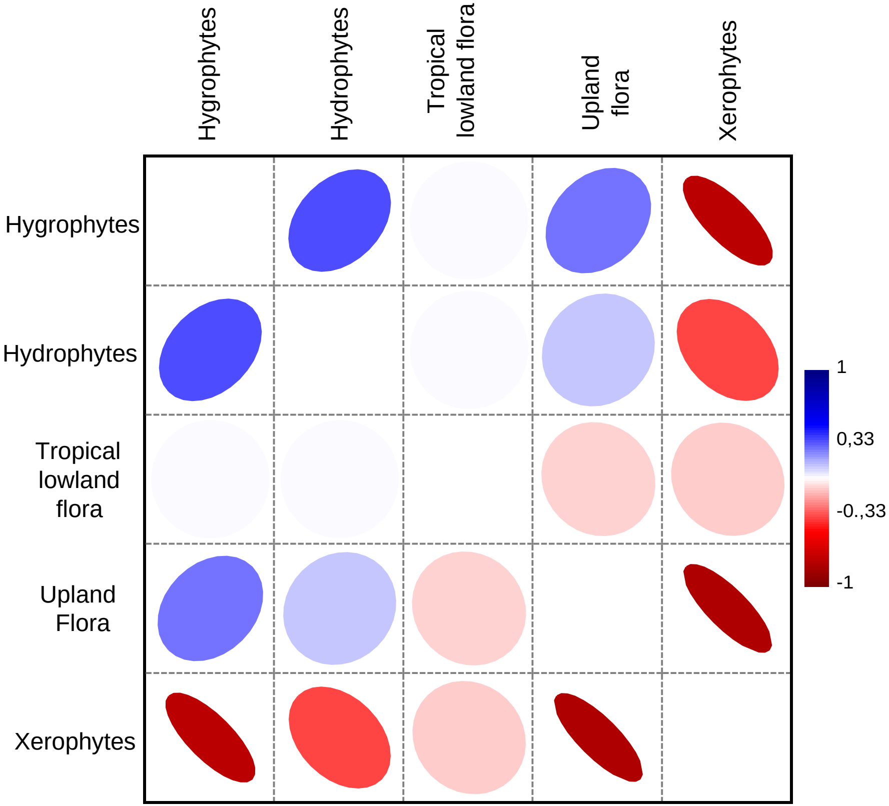

Fig. S7.
Correlation between bioclimatic groups. Pearson correlation analysis for bioclimatic groups showing positive (blue) and negative (red) corr29elation. Ellipsoidal shapes represent more significant values.

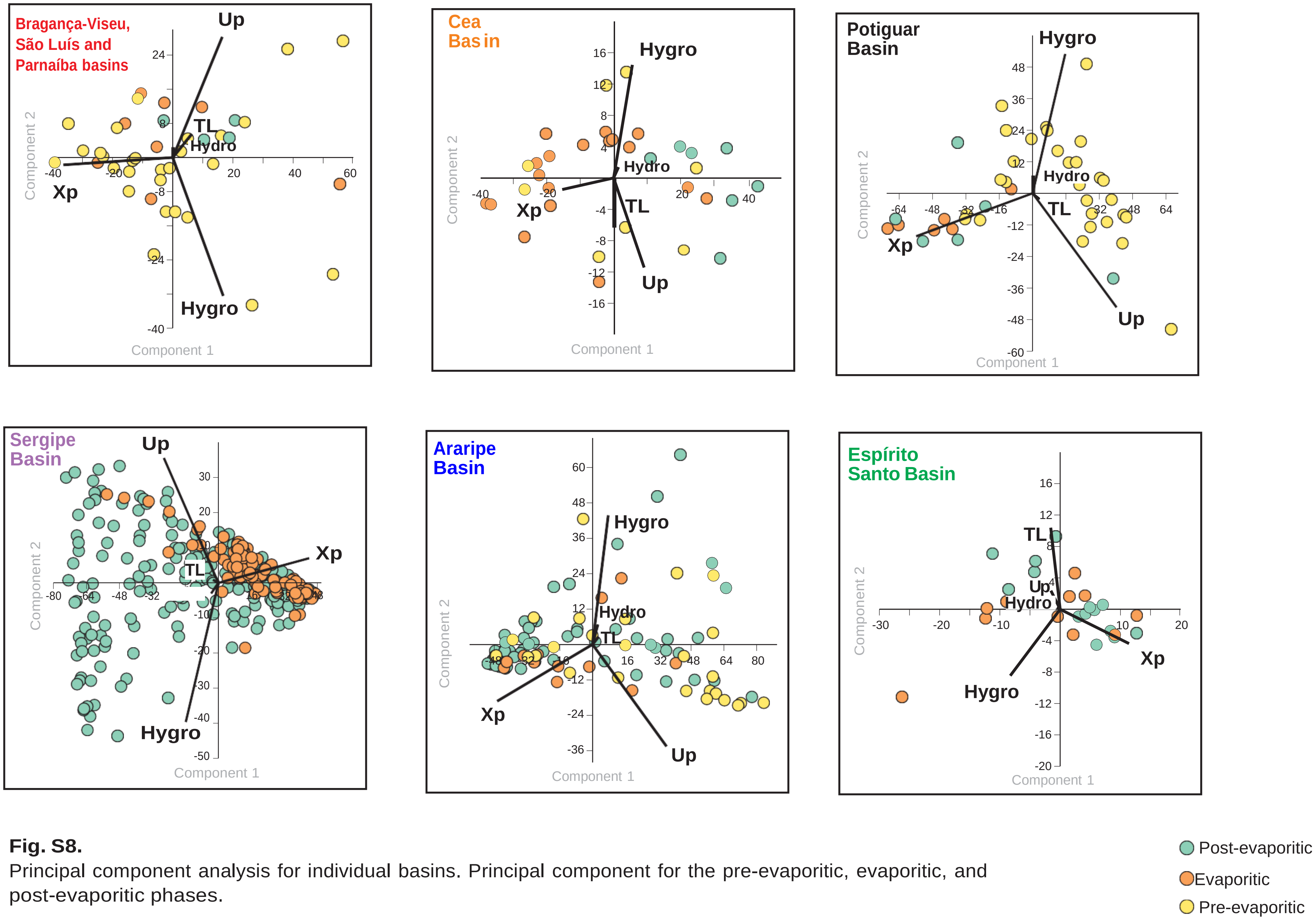

Fig. S8.
Principal component analysis for individual basins. Principal component for the pre-evaporitic, evaporitic, and post-evaporitic phases.

Table S1.
Localities and lithostratigraphy of the studied sections. Number of samples for each climatic phase.

Well names	Well names abraviations	Basins	Lithostratigraphy (Formation/Member)	Interval (m)	Lat (S)	Long (W)	No. total core samples	Paleoclimatic phases		
								Pre-evaporitic	Evaporitic	Post-evaporitic
2-EGST-1-PA	EGST-1	Bragança-Vizeu	Bragança Fm.	676-1872.1	-01:17:55.229	-46:34:55.683	8	8		
2-VN-1-PA	VN-1	Bragança-Vizeu	Bragança Fm.	1287.6-1317.69	-01:06:48.216	-46:40:35.673	4	4		
1-PE-0001-MA	PE-1	São Luís	Codó Fm.	1562-1776.8	-02:22:09.725	-44:57:28.505	4		4	
1-RL-0001-MA	RL-1	São Luís	Codó Fm.	1157.3-1240.3	-02:40:21.105	-45:37:09.065	7	4	3	
2-PR-1-MA	PR-1	São Luís	Codó Fm.	1507.6-1513.1	-01:59:59.070	-45:52:58.477	4			4
1-CI-0001-MA	CI-1	Parnaíba	Codó Fm.	768-907.1	-02:59:54.215	-45:24:30.842	13	12	1	
1-CES-17	CES-17	Ceará	Mundaú Fm.	2095.2-2112.9	-03:06:08.351	-38:51:00.562	8	8		
1-CES-27	CES-27	Ceará	Paracuru Fm.	2119.4-2125	-02:58:30.993	-38:57:24.830	7			7
1-CES-44	CES-44	Ceará	Paracuru Fm./Trairi Mb.	2651.6-2651.9	-02:55:56.004	-38:59:46.335	18		18	
3-RNS-159	RNS-159	Potiguar	Alagamar Fm./Upanema and Galinhos Mbs and Camada Ponta do Tubarão layer	3706.0-4038	-04:31:12.148	-36:44:48.970	41	29	6	6
1-PS-11-CE	PS-11	Araripe	Crato/Ipubi/Romualdo fms.	4.4-167.6	-07:10:33.730	-39:29:00.810	38	19	12	7
2AP-0001-CE	2AP	Araripe	Crato/Romualdo fms.	396.25-488.08	-07:18:09.907	-40:02:34.114	17	8		9
Sobradinho	Sobradinho	Araripe	Romualdo Fm.	79-31	-07:21:34.8	-39:5:54.8	38			38
9-CL-47-SE	CL-47	Sergipe	Riachuelo Fm./Angico Mb.	77.6-217.2	-10:35:59.659	-37:05:28.633	68		18	50
9-GTP-17-SE	GTP-17	Sergipe	Riachuelo Fm./Angico Mb.	18-470.95	-10:39:24.476	-37:10:47.649	102		32	70
9-GTP-24-SE	GTP-24	Sergipe	Riachuelo Fm./Taquari Mb.	13.5-414.95	-10:38:58.453	-37:02:01.698	153		42	111
9-PEI-003-ES	PEI-03	Espírito Santo	Mariricu Fm./Itaúnas Mb. and Regência and São Mateus fms.	890-985	-18:28:41.674	-39:46:00.927	11		6	5
9-PEI-006-ES	PEI-06	Espírito Santo	Mariricu Fm./Itaúnas Mb. and Regência Fm.	569.16-754.3	-18:29:14.606	-39:47:36.995	14		5	9
Total							555	92	147	316

Table S2.
Plant groups, palynomorph taxa, botanical affinities and bioclimatic groups and biomes of the material studied.

Plant Groups	Palynomorph taxa	Botanical affinities	Bioclimatic groups	Biomes
Bryophytes	*Aequitriradites*	Hepaticae	Hygrophyte	Lowland rainforest
	Cingulatisporites	Anthocerotaceae	Hygrophyte	Lowland rainforest
	Cingutriletes	Sphagnaceae	Hygrophyte	Lowland rainforest
	Stereisporites	Sphagnaceae	Hygrophyte	Lowland rainforest
	Triporoletes	Ricciaceae	Hygrophyte	Lowland rainforest
Ferns	*Appendicisporites*	Schizaeales (Anemiaceae?)	Hygrophyte	Lowland rainforest
	Bellispores	?	Hygrophyte	Lowland rainforest
	Biretisporites	Osmundaceae	Hygrophyte	Lowland rainforest
	Birretisporites	?	Hygrophyte	Lowland rainforest
	Cibotiumspora	Matoniaceae	Upland flora	Montane rainforest
	Cicatricosisporites	Schizaeales (Anemiaceae?)	Hygrophyte	Lowland rainforest
	Clavasporites	?	Hygrophyte	Lowland rainforest
	Collarisporites	Marsileaceae	Hydrophyte	Lowland rainforest
	Concavisporites	Gleicheniaceae	Hygrophyte	Lowland rainforest
	Contignisporites	Pteridaceae	Hygrophyte	Lowland rainforest
	Converrucosisporites	Dicksoniaceae	Upland flora	Montane rainforest
	Crybelosporites	Marsileaceae	Hydrophyte	Lowland rainforest
	Cyathidites	Cyatheaceae-Dicksoniaceae	Upland flora	Montane rainforest
	Deltoidospora	Cyatheaceae-Dicksoniaceae	Upland flora	Montane rainforest
	Dictyophyllidites	Matoniaceae	Upland flora	Montane rainforest
	Distaltriangulisporites	Schzaeales (Schizaeaceae?)	Hygrophyte	Lowland rainforest
	Foveomonoletes	Schizaeaceae	Hygrophyte	Lowland rainforest
	Foveotriletes	Schzaeales (Schizaeaceae?)	Hygrophyte	Lowland rainforest
	Gabonisporis	Marsileaceae	Hydrophyte	Lowland rainforest
	Gleicheniidites	Gleicheniaceae	Hygrophyte	Lowland rainforest
	Granulatusporites	Pteridaceae	Hygrophyte	Lowland rainforest
	Klukisporites	Schizaeales (Lygodiaceae?)	Hygrophyte	Lowland rainforest
	Laevigatosporites	Polypodiaceae	Hygrophyte	Lowland rainforest
	Matonisporites	Matoniaceae	Upland flora	Lowland rainforest
	Microapiculate monolete	?	Hygrophyte	Lowland rainforest
	Microfoveolatosporis	Schzaeales (Schizaeaceae?)	Hygrophyte	Lowland rainforest
	Monolete apiculate	?	Hygrophyte	Lowland rainforest
	Monolete gemmate	?	Hygrophyte	Lowland rainforest
	Murospora	?	Hygrophyte	Lowland rainforest
	Osmundacidites	Osmundaceae	Hygrophyte	Lowland rainforest
	Paludites	Marsileaceae	Hydrophyte	Lowland rainforest
	Perisporate trilete	?	Hydrophyte	Lowland rainforest
	Pilosisporites	Schzaeales (Schizaeaceae?)	Hygrophyte	Lowland rainforest
	Plicatella	Schizaeales (Anemiaceae?)	Hygrophyte	Lowland rainforest
	Reticulatosporites	Schzaeales (Schizaeaceae?)	Hygrophyte	Lowland rainforest
	Reticulosporis	Schzaeales (Schizaeaceae?)	Hygrophyte	Lowland rainforest
	Rugulatisporites	Osmundaceae	Hygrophyte	Lowland rainforest
	Schweitzerisporites	?	Hygrophyte	Lowland rainforest
	Scylaspora	?	Hygrophyte	Lowland rainforest
	Todisporites	Osmundaceae	Hygrophyte	Lowland rainforest
	Trilete apiculate	?	Hygrophyte	Lowland rainforest
	Trilete baculate	?	Hygrophyte	Lowland rainforest
	Trilete cingulate	?	Hygrophyte	Lowland rainforest
	Trilete echinate	?	Hygrophyte	Lowland rainforest
	Trilete fenestrate	?	Hygrophyte	Lowland rainforest
	Trilete foveolate	?	Hygrophyte	Lowland rainforest
	Trilete granulate	?	Hygrophyte	Lowland rainforest
	Trilete hamulate	?	Hygrophyte	Lowland rainforest
	Trilete psilate	?	Hygrophyte	Lowland rainforest
	Trilete psilate with labium	?	Hygrophyte	Lowland rainforest
	Trilete psilate zonate	?	Hygrophyte	Lowland rainforest
	Trilete reticulate	?	Hygrophyte	Lowland rainforest
	Trilete rugulate	?	Hygrophyte	Lowland rainforest
	Trilete scrabate	?	Hygrophyte	Lowland rainforest

	Trilete verrucate	?	Hygrophyte	Lowland rainforest
	Trilobosporites	Lygodiaceae	Hygrophyte	Lowland rainforest
	Undulatisporites	Schzaeales (Schizaeaceae?)	Hygrophyte	Lowland rainforest
	Verrucosisporites	Osmundaceae (?)	Hygrophyte	Lowland rainforest
Lycophytes	*Antulsporites*	Selaginellaceae	Hygrophyte	Lowland rainforest
	Apiculatisporis	Selaginellaceae	Hygrophyte	Lowland rainforest
	Camarozonosporites	Lycopodiaceae	Hygrophyte	Lowland rainforest
	Densoisporites	Selaginellaceae	Hygrophyte	Lowland rainforest
	Echinatisporis	Selaginellaceae	Hygrophyte	Lowland rainforest
	Echinatisporites	Selaginellaceae	Hygrophyte	Lowland rainforest
	Echitriletes	Isoetaceae	Hydrophyte	Lowland rainforest
	Foveosporites	Selaginellaceae	Hygrophyte	Lowland rainforest
	Hamulatisporis	Lycopodiaceae	Hygrophyte	Lowland rainforest
	Leptolepidites	Lycopodiaceae	Hygrophyte	Lowland rainforest
	Lycopodiumsporites	Lycopodiaceae	Hygrophyte	Lowland rainforest
	Neoraistrickia	Selaginellaceae	Hygrophyte	Lowland rainforest
	Perotrilites	Selaginellaceae	Hygrophyte	Lowland rainforest
	Retitriletes	Lycopodiaceae	Hygrophyte	Lowland rainforest
	Rugutriletes	Lycopodiaceae	Hygrophyte	Lowland rainforest
	Sestrosporites	Lycopodiaceae	Hygrophyte	Lowland rainforest
	Uvaesporites	Selaginellaceae	Hygrophyte	Lowland rainforest
	Verrutriletes	Selaginellaceae (?)	Hygrophyte	Lowland rainforest
Pteridosperms	*Vitreisporites*	Caytoniaceae	Upland flora	Montane rainforest
Gymnosperms	*Araucariacites*	Araucariaceae	Upland flora	Montane rainforest
	Balmeiopsis	Araucariacites	Upland flora	Montane rainforest
	Bennettitaepollenites	Cycadaceae	Tropical lowland flora	Lowland rainforest
	Callialasporites	Araucariacites/Podocarpaceae	Upland flora	Montane rainforest
	Cavamonocolpites	Cycadaceae	Tropical lowland flora	Lowland rainforest
	Cedripites	Pinaceae	Upland flora	Montane rainforest
	Cingulatipollenites	Araucariaceae	Upland flora	Montane rainforest
	Classopollis	Cheirolepidiaceae	Xerophytes	Tropical xerophytic shrubland
	Complicatisaccus	Coniferae i. sedis	Upland flora	Montane rainforest
	Cycadopites	Cycadaceae	Tropical lowland flora	Lowland rainforest
	Dissacate indeterminate	?	Upland flora	Montane rainforest
	Elateropollenites	Gnetales (Gnetaceae?)	Xerophytes	Tropical xerophytic shrubland
	Equisetosporites	Gnetales (Ephedraceae?)	Xerophytes	Tropical xerophytic shrubland
	Eucommiidites	Gnetales?	Xerophytes	Lowland rainforest
	Exesipollenites	Cupressaceae	Upland flora	Montane rainforest
	Gnetaceaepollenites	Gnetales (Gnetaceae?)	Xerophytes	Tropical xerophytic shrubland
	Inaperturopollenites	Cupressaceae	Upland flora	Montane rainforest
	Monocolpate psilate	?	Tropical lowland flora	Lowland rainforest
	Monosulcites	Cycadaceae	Tropical lowland flora	Lowland rainforest
	Parvisaccites	Podocarpaceae	Upland flora	Montane rainforest
	Regalipollenites	Gnetales (Ephedraceae?)	Xerophytes	Tropical xerophytic shrubland
	Rugubivesiculites	Podocarpaceae	Upland flora	Montane rainforest
	Sergipea	Gnetales	Xerophytes	Tropical xerophytic shrubland
	Singhia	Gnetales (Ephedraceae?)	Xerophytes	Tropical xerophytic shrubland
	Spheripollenites	Cupressaceae	Upland flora	Montane rainforest
	Steevesipollenites	Gnetales (Gnetaceae?)	Xerophytes	Tropical xerophytic shrubland
	Uesuguipollenites	Cupressaceae	Upland flora	Montane rainforest
	Afropollis	?	Tropical lowland flora	Lowland rainforest
	Arecipites	Monocots (Arecaceae?)	Tropical lowland flora	Lowland rainforest
	Brenneripollis	Chloranthaceae	Tropical lowland flora	Lowland rainforest
	Clavatipollenites	Chloranthaceae	Tropical lowland flora	Lowland rainforest
	Cornetipollis	?	Tropical lowland flora	Lowland rainforest
	Cretacaeiporites	Trimeniaceae?	Tropical lowland flora	Lowland rainforest
	Dejaxpollenites	?	Tropical lowland flora	Lowland rainforest
	Dicolpate indeterminate	?	Tropical lowland flora	Lowland rainforest
	Monocolpate intrareticulate	?	Tropical lowland flora	Lowland rainforest

Angiosperms	Monocolpate reticulate	?	Tropical lowland flora	Lowland rainforest
	Monocolpate verrucate	?	Tropical lowland flora	Lowland rainforest
	Monocolpopollenites	Monocots (Arecaceae?)	Tropical lowland flora	Lowland rainforest
	Monosulcate reticulate	?	Tropical lowland flora	Lowland rainforest
	Psiladicolpites	Monocots (Liliaceae?)	Tropical lowland flora	Lowland rainforest
	Psilatricolpites	Eudicots (Euphorbiaceae?)	Tropical lowland flora	Lowland rainforest
	Quadricolpites	?	Tropical lowland flora	Lowland rainforest
	Retimonocolpites	Monocots (Arecaceae?)	Tropical lowland flora	Lowland rainforest
	Retiquadricolpites	?	Tropical lowland flora	Lowland rainforest
	Retitricolpites	?	Tropical lowland flora	Lowland rainforest
	Rousea	Eudicots (Flacourtiaceae?)	Tropical lowland flora	Lowland rainforest
	Schrankipollis	?	Tropical lowland flora	Lowland rainforest
	Stellatopolis	?	Tropical lowland flora	Lowland rainforest
	Striatopollis	Solanaceae	Tropical lowland flora	Lowland rainforest
	Tetracolpate reticulate	?	Tropical lowland flora	Lowland rainforest
	Tricolpate clavate	?	Tropical lowland flora	Lowland rainforest
	Tricolpate psilate	?	Tropical lowland flora	Lowland rainforest
	Tricolpate reticulate	?	Tropical lowland flora	Lowland rainforest
	Tricolpites	Eudicots	Tropical lowland flora	Lowland rainforest
	Tricolporate psilate	?	Tropical lowland flora	Lowland rainforest
	Trisectoris	Illiciaceae	Tropical lowland flora	Lowland rainforest

Table S3.
Percentage abundance of bioclimatic groups and diversity, Fs/X and marine elements for samples from the Bragança-Viseu, São Luís, and Parnaíba basins. Legend: HG = hygrophytes; HD = hydrophytes; TLF = tropical lowland flora; UF = upland flora; XP = xerophytes; H' = diversity; Fs/X = spores/xerophytes.

Lithostratigraphy	Sections	Depth (m)	HG	HD	TLF	UF	XP	H'	Fs/X	Marine
Codó Formation	2-PR-1-MA	1507.6	15.7	1.5	31.0	14.2	37.6	2.26	0.31	0
		1509.7	19.6	0.5	8.2	29.4	42.3	2.19	0.32	0
		1510.6	15.4	0.0	22.0	15.4	47.3	1.88	0.25	0
		1513.1	6.6	2.0	20.2	13.1	58.1	2.04	0.13	0
	1-PE-1-MA	1562.0	44.4	0.0	8.7	33.2	13.8	2.59	0.76	0
		1566.0	14.4	0.6	2.2	30.0	52.8	2.21	0.22	0
		1568.5	4.2	0.0	20.8	16.7	58.3	1.99	0.07	0
		1570.0	4.1	0.0	2.4	24.1	69.4	1.90	0.06	0
	1-RL-1-MA	1173.5	1.6	0.0	23.8	6.3	68.3	2.22	0.02	0
		1174.1	12.0	4.0	10.0	12.0	62.0	2.06	0.21	0
		1175.5	20.0	0.0	14.5	1.8	63.6	2.63	0.24	0
		1235.25	18.8	1.6	10.2	7.8	61.7	2.57	0.25	1
		1237.0	19.8	0.0	4.9	14.2	61.1	2.61	0.24	0
		1239.5	27.0	1.6	6.3	6.3	58.7	1.85	0.33	0
		1240.3	11.2	0.5	13.8	5.9	68.6	1.91	0.15	0
	1-CI-1-MA	820.6	6.5	0.0	15.7	0.0	77.8	1.78	0.08	0
		834.5	3.7	0.0	3.7	0.0	92.6	1.30	0.04	0
		836.0	0.0	0.0	0.0	10.2	89.8	1.66	0.00	0
		837.0	14.7	2.7	6.0	48.9	27.7	2.28	0.18	0
		838.0	15.8	0.0	1.5	9.8	72.9	1.74	0.06	0
		845.0	4.8	0.0	1.4	23.3	70.5	2.07	0.33	0
		855.0	5.2	0.0	5.2	5.2	84.5	1.10	0.13	0
		855.9	10.8	0.6	10.1	3.8	74.7	2.11	0.38	0
		857.6	21.6	2.6	4.2	31.6	40.0	2.64	0.10	1
		866.55	8.6	0.5	5.6	6.6	78.8	2.27	0.25	1
		866.65	16.9	1.6	4.4	21.3	55.7	2.26	0.21	0
		867.8	16.0	0.0	16.6	7.1	60.4	2.83	0.39	0
		888.75	19.3	0.0	12.9	22.9	45.0	2.28	0.30	0
Bragança Formation	2-VN-1-PA	1287.9	55.0	0.0	0.0	5.0	40.0	1.56	0.58	0
		1289.88	18.4	0.0	5.3	18.4	57.9	1.94	0.24	0
		1315.7	6.7	0.6	1.2	15.3	76.1	1.57	0.09	0
		1317.69	33.3	0.0	0.0	0.0	66.7	1.29	0.33	0
	2-EGST-1-PA	676.44	19.2	0.5	14.8	54.4	11.0	2.54	0.64	0
		732.3	30.1	2.4	3.0	8.4	56.0	2.65	0.37	0
		733.3	8.1	0.0	4.3	7.6	80.0	1.48	0.09	0
		735.3	13.7	3.0	0.5	12.2	70.6	1.97	0.19	0
		1017.7	25.6	1.2	0.6	22.6	50.0	2.42	0.35	0
		1789.1	19.0	0.0	1.7	6.3	73.0	1.83	0.21	0
		1791.0	26.7	0.0	4.4	6.7	62.2	1.92	0.30	0
		1846.0	57.1	0.0	14.3	14.3	14.3	1.95	0.80	0

Table S4.
Percentage abundance of bioclimatic groups and diversity, Fs/X and marine elements for samples from the Ceará Basin. Legend: HG = hygrophytes; HD = hydrophytes; TLF = tropical lowland flora; UF = upland flora; XP = xerophytes; H' = diversity; Fs/X = spores/xerophytes.

Lithostratigraphy	Sections	Depth (m)	HG	HD	TLF	UF	XP	H'	Fs/X	Marine
Paracuru Formation	1-CES-27	2644.4	31.1	1.1	3.2	41.1	23.7	3.10	0.58	0
		2644.8	28.0	1.0	4.0	28.5	38.5	3.19	0.43	0
		2646.15	26.8	1.1	5.3	37.9	28.9	2.97	0.49	0
		2646.6	28.0	0.5	2.6	27.0	41.8	2.75	0.41	0
		2648.6	24.0	0.5	3.1	23.4	49.0	2.96	0.33	0
		2648.75	21.0	0.0	2.6	42.6	33.8	2.72	0.38	0
		2650.0	32.6	0.5	0.0	35.3	31.6	2.66	0.51	0
Paracuru Formation - Trairi Member	1-CES-44	2651.6	5.1	0.0	0.0	4.3	90.6	1.07	0.05	0
		2651.9	5.3	0.9	11.5	22.1	60.2	1.47	0.09	0
		2652.5	10.2	0.0	3.1	12.6	74.0	1.41	0.12	0
		2653.3	4.6	0.0	3.1	11.3	80.9	1.23	0.05	0
		2654.6	21.9	0.0	10.4	16.1	51.6	1.64	0.30	0
		2654.8	22.2	1.5	1.5	16.5	58.2	1.74	0.29	0
		2655.3	24.7	0.0	6.8	17.9	50.5	2.03	0.33	0
		2655.6	25.0	0.5	4.7	34.4	35.4	1.99	0.42	0
		2656.3	15.3	0.0	1.6	9.0	74.1	1.47	0.17	0
		2656.7	24.1	1.6	1.6	31.4	41.4	2.08	0.38	0
		2657.65	22.7	0.0	2.6	14.9	59.8	1.73	0.28	0
		2658.8	22.4	0.0	1.6	16.7	59.4	1.86	0.27	0
		2659.5	19.1	0.5	2.6	12.4	65.5	1.70	0.23	0
		2660.6	11.9	0.0	4.6	7.7	75.8	1.31	0.14	0
		2661.3	3.7	1.0	6.8	1.6	86.9	1.43	0.05	0
		2661.8	12.1	3.5	1.5	6.6	76.3	1.51	0.17	0
		2662.5	16.6	0.5	3.1	5.7	74.1	1.53	0.19	0
		2663.5	11.1	2.5	2.0	10.6	73.9	1.74	0.16	0
Mundaú Formation	1-CES-17	2665.2	8.5	0.6	8.5	21.5	61.0	2.26	0.13	0
		2666.2	15.0	0.0	3.6	25.4	56.0	2.64	0.21	0
		2668.1	25.0	6.5	1.9	9.3	57.4	2.98	0.35	0
		2669.7	11.8	1.5	1.5	6.2	79.0	2.19	0.14	0
		2670.8	19.4	21.4	2.6	24.5	32.1	2.49	0.56	0
		2672.75	29.2	3.6	2.4	11.9	53.0	2.64	0.38	0
		2673.5	16.1	5.4	2.7	34.8	41.1	2.78	0.34	0
		2676.45	8.7	1.9	3.1	6.8	79.5	2.05	0.12	0

Table S5.
Percentage abundance of bioclimatic groups and diversity, Fs/X and marine elements for samples from the Potiguar Basin. Legend: HG = hygrophytes; HD = hydrophytes; TLF = tropical lowland flora; UF = upland flora; XP = xerophytes; H’ = diversity; Fs/X = spores/xerophytes.

Lithostratigraphy	Section	Depth (m)	HG	HD	TLF	UF	XP	H'	Fs/X	Marine
Alagamar Formation - Upanema Member	3-RNS-159	3706	0.0	0.0	0.0	12.5	87.5	1.15	0	0
		3771	3.8	0.0	0.0	0.0	96.2	0.43	0.04	8
		3793	11.1	0.0	16.7	16.7	55.6	1.69	0.17	0
		3794	9.1	0.0	0.0	72.7	18.2	0.76	0.33	0
		3796.6	3.4	0.7	1.4	21.1	73.5	1.69	0.05	4
Alagamar Formation - Camada Ponta dfo Tubarão (layer)		3803.4	33.3	0.0	0.0	0.0	66.7	1.15	0.33	0
		3813.5	7.5	0.9	2.8	12.3	76.4	1.15	0.10	0
		3816.4	0.0	0.0	0.0	0.0	100.0	0	0.00	0
		3838	2.0	0.5	0.0	2.0	95.5	0.36	0.03	0
		3840.8	6.8	0.0	0.0	17.8	75.3	1.01	0.08	0
		3843.6	25.0	0.0	0.0	25.0	50.0	1.04	0.33	0
Alagamar Formation - Upanema Member		3845.8	4.5	0.0	0.0	13.1	82.4	0.77	0.05	0
		3851.2	15.5	3.4	0.0	55.2	25.9	1.69	0.42	0
		3854.8	39.3	4.8	2.4	17.3	36.3	2.29	0.55	0
		3856.34	42.9	14.3	0.0	0.0	42.9	1.55	0.57	0
		3856.72	42.1	0.0	0.0	10.5	47.4	1.91	0.47	0
		3861.24	7.1	7.1	7.1	14.3	64.3	1.83	0.18	0
		3861.96	28.4	10.8	1.4	16.2	43.2	2.45	0.48	0
		3863.25	41.7	7.5	6.7	16.7	27.5	2.50	0.64	0
		3865.48	39.7	14.3	1.6	17.5	27.0	2.23	0.67	0
		3874.97	14.3	28.6	0.0	14.3	42.9	1.55	0.50	0
		3877.91	70.6	0.0	0.0	17.6	11.8	1.86	0.86	0
		3878.49	21.0	11.0	1.5	49.0	17.5	2.52	0.65	0
		3882.95	39.1	0.0	0.0	34.8	26.1	2.26	0.60	0
		3883.31	46.7	0.0	0.0	33.3	20.0	1.93	0.70	0
		3885.35	26.7	0.0	0.0	20.0	53.3	1.69	0.33	0
		3886.04	0.0	0.0	0.0	100.0	0.0	-	0.00	0
		3888.95	35.9	4.0	0.5	45.5	14.1	2.66	0.74	0
		3890.92	31.3	1.5	1.5	53.8	11.8	2.22	0.74	0
		3891	35.5	4.0	0.5	47.0	13.0	2.39	0.75	0
		3891.6	18.5	4.5	0.5	66.0	10.5	1.962	0.69	0
		3892.21	28.3	1.0	1.5	60.6	8.6	2.18	0.77	0
		3893.27	29.6	0.0	0.0	48.1	22.2	1.62	0.57	0
		3895.1	31.0	6.0	0.0	41.0	22.0	2.16	0.63	0
		3895.45	21.4	0.9	0.9	54.5	22.3	2.27	0.50	0
		3896.9	40.0	0.0	0.0	36.7	23.3	1.95	0.63	0
		3898.56	28.3	1.3	0.0	62.3	8.2	1.65	0.78	0
		3900.27	24.4	2.2	0.0	57.8	15.6	1.54	0.63	0

		3901.39	36.9	9.5	1.2	26.2	26.2	2.61	0.64	0
		3955.2	12.4	0.0	0.0	23.4	64.2	1.617	0.16	0
		4038	9.6	3.6	0.0	18.1	68.7	1.853	0.16	0

Table S6.
Percentage abundance of bioclimatic groups and diversity, Fs/X and marine elements for samples from the Sergipe Basin. Legend: HG = hygrophytes; HD = hydrophytes; TLF = tropical lowland flora; UF = upland flora; XP = xerophytes; H' = diversity; Fs/X = spores/xerophytes.

Lithostratigraphy	Sections	Depth (m)	HG	HD	TLF	UF	XP	H'	Fs/X	Marine
Riachuelo Formation – Taquari Member	9-GTP-24-SE	13.25	9.68	0.00	0.00	70.97	19.35	1.69	0.68	41
		15.78	12.12	0.00	0.00	3.03	84.85	0.59	0.15	80
		16.00	14.29	0.00	0.00	8.57	77.14	1.20	0.21	64
		16.45	1.72	0.00	0.86	11.21	86.21	0.57	0.03	18
		16.65	8.33	0.00	0.00	58.33	33.33	1.24	0.43	43
		17.30	5.56	0.00	0.00	20.37	74.07	0.93	0.11	5
		18.63	3.70	0.00	3.70	59.26	33.33	1.63	0.44	125
		19.24	6.67	0.00	0.00	10.00	83.33	0.67	0.11	5
		19.35	5.00	0.00	0.00	40.00	55.00	1.02	0.15	26
		19.85	9.09	0.00	0.00	22.73	68.18	0.94	0.14	22
		20.53	0.00	0.00	0.00	32.00	68.00	0.82	0.00	26
		20.85	16.67	0.00	0.00	16.67	66.67	1.33	0.33	69
		22.80	8.00	0.00	0.00	32.00	60.00	1.16	0.17	82
		22.88	40.57	0.94	0.00	44.34	14.15	2.03	0.82	104
		23.55	15.38	0.00	0.00	61.54	23.08	1.30	0.50	25
		24.05	0.00	0.00	0.00	19.64	80.36	0.59	0.04	9
		26.23	3.31	1.65	5.79	29.75	59.50	1.15	0.09	44
		26.83	7.35	0.74	2.21	16.91	72.79	1.00	0.11	69
		28.00	7.41	0.00	3.70	44.44	44.44	1.48	0.25	185
		30.60	4.38	0.00	1.46	44.53	49.64	1.24	0.12	27
		32.25	8.00	0.00	16.00	48.00	28.00	1.08	0.22	178
		33.20	6.78	0.00	13.56	25.42	54.24	1.54	0.14	155
		35.95	8.11	0.00	0.00	35.14	56.76	1.33	0.16	73
		37.55	3.33	0.00	10.00	6.67	80.00	0.57	0.04	178
		40.10	2.94	5.88	8.82	32.35	50.00	1.64	0.16	190
		42.25	8.20	0.00	3.28	29.51	59.02	1.22	0.16	124
		42.65	26.09	0.00	4.35	26.09	43.48	1.50	0.41	192
		44.15	0.00	0.00	8.33	27.78	63.89	1.34	0.00	7
		46.35	13.16	5.26	7.89	10.53	63.16	1.91	0.24	191
		47.90	9.52	0.00	3.17	30.16	57.14	1.34	0.18	88
		48.60	4.21	0.00	4.21	10.53	81.05	0.83	0.06	117
		49.70	20.00	0.00	6.00	28.00	46.00	2.01	0.38	160
		50.60	10.83	1.67	1.67	35.00	50.83	1.54	0.28	87
		51.80	30.34	1.12	8.99	34.83	24.72	2.45	0.62	126
		52.35	10.64	0.00	0.00	54.26	35.11	1.27	0.26	114
		54.50	12.00	0.00	0.00	82.00	6.00	0.94	0.79	109
		56.10	11.11	0.00	0.00	72.22	16.67	1.43	0.67	169
		56.95	10.87	0.00	2.17	21.74	65.22	1.27	0.19	15

		57.60	12.50	0.00	0.00	70.83	16.67	1.18	0.60	109
		57.80	0.00	0.00	0.00	54.55	45.45	1.11	0.17	79
		58.70	25.49	0.00	0.00	47.06	27.45	1.75	0.58	115
		59.30	1.05	0.00	0.00	34.74	64.21	0.93	0.06	40
		60.70	5.00	0.00	0.00	50.00	45.00	1.57	0.38	185
		65.45	40.00	0.00	0.00	33.33	26.67	1.90	0.64	250
		65.90	2.22	0.00	0.00	53.33	44.44	1.17	0.20	32
		67.00	22.92	0.00	0.00	68.75	8.33	1.25	0.80	44
		67.40	20.00	0.00	0.00	60.00	20.00	1.13	0.50	72
		71.60	23.53	0.00	0.00	58.82	17.65	1.50	0.68	56
		72.25	44.44	0.00	0.00	16.67	38.89	1.75	0.59	139
		75.40	5.10	0.00	0.00	59.18	35.71	0.94	0.13	70
		77.20	6.82	0.00	0.00	20.45	72.73	0.85	0.11	49
		80.40	19.35	3.23	0.00	30.65	46.77	1.76	0.37	139
		82.55	10.98	0.00	0.00	36.59	52.44	1.41	0.27	97
		83.00	16.67	0.00	0.00	12.50	70.83	1.31	0.26	102
		84.90	4.44	0.00	0.00	29.63	65.93	1.03	0.12	58
		86.70	12.90	0.00	0.00	29.03	58.06	1.46	0.31	157
		88.40	5.26	0.66	0.66	6.58	86.84	0.69	0.07	50
		91.25	20.90	0.56	6.78	38.42	33.33	1.74	0.43	67
		94.35	6.87	0.00	0.76	24.43	67.94	1.14	0.09	121
		97.65	24.14	0.00	6.90	31.03	37.93	1.70	0.39	160
		98.20	17.82	0.00	4.95	29.70	47.52	1.55	0.27	54
		99.20	12.63	0.00	5.79	33.68	47.89	1.75	0.24	42
		101.70	27.40	0.00	6.16	23.29	43.15	1.80	0.42	63
		103.15	12.02	0.55	1.09	26.78	59.56	1.45	0.19	68
		108.10	1.60	0.00	1.60	8.80	88.00	0.55	0.04	88
		109.40	22.92	0.00	3.13	9.38	64.58	1.50	0.27	118
		110.60	7.93	0.61	1.22	6.71	83.54	0.90	0.09	76
		112.70	7.80	0.00	2.13	21.99	68.09	1.11	0.13	49
		119.50	6.63	0.00	1.81	8.43	83.13	0.83	0.09	59
		120.60	9.23	0.00	3.08	0.00	87.69	0.48	0.10	138
		122.85	0.00	0.00	0.00	2.33	97.67	0.22	0.02	90
		124.40	4.82	0.00	3.61	8.43	83.13	0.81	0.08	124
		124.67	10.59	0.00	2.35	11.76	75.29	0.91	0.15	162
		128.10	3.26	0.00	3.26	7.07	86.41	0.69	0.04	69
		130.55	2.74	1.37	1.37	15.75	78.77	0.87	0.12	223
		134.45	5.17	0.00	3.45	7.76	83.62	0.86	0.06	177
		135.75	20.83	0.00	4.17	41.67	33.33	1.78	0.53	190
		146.10	12.93	0.86	4.31	50.86	31.03	1.89	0.45	123
		157.15	20.63	0.53	4.76	44.44	29.63	2.08	0.48	31
		160.35	18.02	0.00	4.50	45.05	32.43	1.91	0.48	184
		167.25	12.05	1.20	1.20	39.76	45.78	1.64	0.25	267
		170.43	20.16	0.00	2.33	31.78	45.74	1.78	0.43	243
		178.56	16.42	0.00	5.97	23.88	53.73	1.54	0.27	90

		182.06	9.40	0.00	1.71	13.68	75.21	1.03	0.13	138
		182.60	15.97	0.00	0.69	7.64	75.69	1.01	0.20	65
		189.80	4.82	0.00	4.82	24.10	66.27	1.24	0.08	29
		190.75	4.42	0.00	1.77	19.47	74.34	1.06	0.07	215
		200.68	3.45	0.00	0.00	5.17	91.38	0.39	0.05	27
		202.37	3.42	0.00	0.00	6.85	89.73	0.56	0.06	36
		205.35	7.74	0.00	0.65	9.03	82.58	0.72	0.10	49
		207.80	6.78	0.00	0.00	22.03	71.19	1.32	0.22	20
		211.85	6.00	1.00	3.50	14.50	75.00	1.36	0.12	12
		214.75	3.49	0.00	0.58	4.65	91.28	0.59	0.05	166
		217.00	5.05	0.00	0.00	8.08	86.87	0.72	0.07	4
		222.00	2.12	0.00	0.53	4.23	93.12	0.43	0.03	22
		222.25	3.45	0.00	0.00	4.60	91.95	0.45	0.04	38
		224.60	1.88	0.00	0.63	1.25	96.25	0.23	0.03	5
		227.10	5.99	0.00	1.84	3.69	88.48	0.69	0.07	42
		232.90	2.08	0.00	0.00	4.17	93.75	0.32	0.02	10
		237.60	2.22	0.00	0.00	3.89	93.89	0.35	0.03	30
		237.70	4.81	0.00	0.00	1.92	93.27	0.43	0.06	1
		239.75	2.53	0.00	0.51	7.07	89.90	0.51	0.04	10
		240.65	4.10	0.00	0.00	3.08	92.82	0.40	0.05	69
		241.25	0.00	0.00	0.00	10.00	90.00	0.33	0.10	41
		243.95	0.51	0.00	0.00	3.57	95.92	0.23	0.01	12
		247.00	2.48	0.00	0.50	2.97	94.06	0.41	0.04	2
		249.10	2.06	0.00	0.52	1.03	96.39	0.25	0.03	1
		252.60	2.59	0.00	2.07	3.63	91.71	0.45	0.04	9
		254.60	0.00	0.00	2.13	4.26	93.62	0.28	0.00	20
		255.65	2.23	0.00	0.00	1.12	96.65	0.20	0.02	30
		256.10	0.55	0.00	1.10	3.30	95.05	0.28	0.03	71
Riachuelo Formation – Angico Member	9-GTP-17-SE	18.00	15.63	0.00	1.56	33.85	48.96	1.62	0.39	8
		34.75	8.33	0.00	1.04	74.48	16.15	0.99	0.42	0
		57.30	5.24	0.00	0.52	75.39	18.85	0.92	0.31	9
		58.95	5.19	0.00	1.30	57.14	36.36	0.95	0.14	46
		61.05	13.95	0.00	0.00	30.23	55.81	1.13	0.23	53
		61.70	9.68	0.00	0.00	35.48	54.84	0.93	0.15	83
		65.80	1.61	0.00	0.00	29.03	69.35	0.80	0.06	14
		70.60	9.57	0.00	1.06	40.43	48.94	1.23	0.23	12
		82.15	17.99	0.00	0.72	71.94	9.35	1.34	0.75	61
		85.70	9.42	0.00	0.52	80.63	9.42	1.05	0.67	9
		93.90	13.75	0.00	1.25	71.25	13.75	1.56	0.76	40
		96.00	5.61	0.00	0.51	47.96	45.92	1.24	0.20	4
		100.90	15.18	2.62	0.52	29.32	52.36	1.44	0.42	8
		103.30	0.89	0.89	0.89	70.54	26.79	1.11	0.27	88
		125.50	20.00	0.00	1.00	52.00	27.00	1.57	0.52	93
		131.70	2.94	0.00	0.59	20.00	76.47	0.86	0.07	30
		137.70	2.84	0.57	1.70	21.02	73.86	1.02	0.12	23

		141.60	4.50	0.00	0.00	28.50	67.00	0.83	0.08	0
		143.97	24.12	0.00	0.50	32.66	42.71	1.67	0.42	0
		155.70	6.03	0.00	0.86	12.93	80.17	0.70	0.10	17
		158.30	10.92	0.00	0.00	42.53	46.55	1.25	0.30	2
		159.43	6.00	0.00	0.00	11.00	83.00	0.70	0.14	0
		167.70	19.10	0.00	1.51	20.60	58.79	1.58	0.32	1
		174.06	5.46	0.00	0.55	10.93	83.06	0.69	0.09	17
		182.10	9.24	0.00	0.00	63.59	27.17	1.23	0.31	3
		184.00	3.02	0.00	1.01	7.04	88.94	0.54	0.05	1
		209.50	2.00	0.00	0.00	17.00	81.00	0.65	0.03	0
		210.55	8.94	0.00	0.00	25.70	65.36	1.08	0.15	4
		215.05	2.53	0.00	0.51	6.57	90.40	0.48	0.06	2
		216.60	2.60	0.00	1.56	17.71	78.13	0.85	0.06	8
		218.10	3.63	0.00	0.00	6.74	89.64	0.47	0.05	2
		225.15	7.54	0.00	0.00	7.54	84.92	0.67	0.09	1
		230.50	4.48	0.00	1.49	14.18	79.85	0.82	0.12	9
		233.27	7.22	0.00	0.56	16.11	76.11	0.87	0.13	20
		233.70	5.00	0.00	0.50	15.00	79.50	0.78	0.08	0
		242.05	2.01	0.00	2.01	12.56	83.42	0.69	0.03	1
		244.35	10.50	0.00	0.00	41.50	48.00	1.19	0.21	0
		246.75	16.16	0.00	0.51	22.73	60.61	1.23	0.23	2
		248.40	14.36	0.00	0.00	13.85	71.79	1.02	0.19	0
		250.73	8.54	0.00	0.00	14.57	76.88	0.87	0.12	1
		252.75	5.17	0.00	0.00	13.79	81.03	0.69	0.10	26
		254.83	3.02	0.00	0.00	12.06	84.92	0.54	0.03	1
		255.57	2.55	0.00	0.00	13.78	83.67	0.56	0.04	4
		257.65	5.73	0.00	1.04	16.67	76.56	0.86	0.08	8
		259.87	4.04	0.00	0.00	16.16	79.80	0.69	0.07	2
		261.70	2.67	0.00	0.00	8.02	89.30	0.47	0.05	13
		263.32	2.56	0.00	0.51	14.36	82.56	0.64	0.04	0
		265.78	4.17	0.00	0.00	25.00	70.83	0.87	0.09	8
		268.40	2.65	0.00	0.00	20.35	76.99	0.68	0.05	6
		271.42	1.02	0.00	0.00	6.12	92.86	0.31	0.04	3
		273.20	2.61	0.00	0.00	9.15	88.24	0.47	0.04	3
		275.72	3.23	0.00	0.54	10.75	85.48	0.58	0.05	14
		277.75	6.62	0.00	0.00	9.56	83.82	0.68	0.10	1
		283.83	1.60	0.00	0.00	14.97	83.42	0.63	0.03	13
		285.83	1.41	0.00	0.70	6.34	91.55	0.43	0.02	58
		287.02	1.32	0.00	1.32	7.28	90.07	0.43	0.02	49
		292.25	3.45	0.00	0.57	16.09	79.89	0.73	0.08	26
		294.75	2.11	0.00	1.05	8.42	88.42	0.54	0.03	10
		298.30	1.52	0.51	0.00	6.60	91.37	0.42	0.04	3
		300.93	2.08	0.00	0.00	9.38	88.54	0.53	0.03	7
		302.25	0.00	0.00	0.50	12.06	87.44	0.44	0.01	1
		304.18	1.05	0.00	0.53	14.74	83.68	0.57	0.01	10

		306.00	1.58	0.00	0.00	6.84	91.58	0.35	0.02	10
		309.05	1.61	0.00	0.00	3.76	94.62	0.27	0.02	14
		315.23	2.02	0.00	0.00	3.03	94.95	0.26	0.03	2
		317.12	3.17	0.00	0.00	3.70	93.12	0.34	0.04	11
		318.00	1.68	0.00	0.00	4.47	93.85	0.29	0.02	0
		322.95	4.57	0.00	0.51	6.60	88.32	0.50	0.05	3
		325.80	0.00	0.00	0.00	0.65	99.35	0.04	0.00	6
		327.31	1.16	0.00	0.58	2.91	95.35	0.27	0.02	28
	9-CL-47-SE	77.46	42.01	3.55	0.59	46.15	7.69	3.00	0.86	6
		79.48	58.02	4.32	0.62	30.86	6.17	2.50	0.91	31
		87.20	45.39	2.63	0.00	29.61	22.37	2.90	0.68	6
		89.00	47.79	2.21	0.74	29.41	19.85	2.53	0.72	24
		89.90	57.64	6.25	0.00	20.14	15.97	2.71	0.80	51
		90.67	43.11	2.40	1.20	48.50	4.79	2.89	0.91	11
		92.18	40.36	4.82	1.20	39.76	13.86	2.92	0.77	17
		93.54	38.92	4.86	2.16	47.57	6.49	2.95	0.87	9
		95.08	54.55	4.24	1.82	31.52	7.88	2.80	0.88	17
		95.73	42.69	5.26	1.17	41.52	9.36	3.13	0.84	23
		96.30	51.30	3.63	0.52	40.41	4.15	2.77	0.93	4
		97.00	27.59	6.32	2.30	53.45	10.34	3.29	0.77	14
		97.50	52.36	3.14	0.52	40.31	3.66	2.68	0.94	2
		98.85	56.14	5.26	0.00	33.33	5.26	2.80	0.92	81
		99.30	47.85	1.23	1.23	46.01	3.68	2.87	0.93	24
		103.70	44.59	1.27	1.27	47.13	5.73	2.92	0.89	38
		104.88	24.18	3.27	1.31	64.05	7.19	3.02	0.79	34
		105.56	45.87	4.59	0.00	37.61	11.93	2.64	0.81	92
		107.08	17.71	1.04	0.52	44.79	35.94	3.02	0.34	0
		107.43	16.30	0.54	1.09	65.22	16.85	2.95	0.50	1
		108.47	15.50	1.00	1.50	39.50	42.50	2.79	0.28	1
		112.68	51.96	0.56	0.00	38.55	8.94	2.96	0.85	9
		114.29	29.14	9.71	1.71	49.71	9.71	3.12	0.80	1
		114.86	27.13	5.32	0.53	62.77	4.26	3.15	0.88	8
		115.60	32.79	1.64	1.09	61.75	2.73	3.01	0.93	14
		116.40	36.36	1.07	1.07	56.15	5.35	3.01	0.88	1
		117.32	36.67	1.11	1.67	56.11	4.44	3.10	0.90	1
		123.35	20.27	2.70	8.11	63.51	5.41	2.89	0.81	121
		124.48	42.11	7.89	5.26	42.11	2.63	3.04	0.95	172
		125.42	34.38	6.25	3.13	46.88	9.38	2.74	0.81	182
		125.98	55.90	8.07	1.24	32.30	2.48	2.99	0.96	36
		126.24	52.66	4.73	1.18	39.64	1.78	3.12	0.97	26
		127.92	57.30	3.37	1.69	33.71	3.93	2.93	0.94	11
		128.77	39.64	4.73	1.78	44.97	8.88	2.78	0.83	20
		130.10	39.24	3.80	1.90	40.51	14.56	2.39	0.75	31
		131.90	30.48	3.74	3.21	55.61	6.95	3.34	0.83	15
		132.67	35.51	5.80	0.72	43.48	14.49	3.31	0.74	62

		133.30	30.71	2.14	3.57	38.57	25.00	3.46	0.57	51
		136.80	61.62	3.78	0.54	29.19	4.86	3.17	0.93	9
		138.80	22.16	6.49	2.16	40.54	28.65	3.22	0.50	3
		145.75	16.20	1.12	0.56	15.08	67.04	2.63	0.21	5
		146.69	5.06	1.90	0.00	12.66	80.38	1.77	0.08	9
		147.70	4.65	2.91	1.74	11.63	79.07	1.87	0.09	7
		152.69	1.27	0.63	0.00	3.80	94.30	1.50	0.02	9
		153.83	10.53	4.61	1.32	25.00	58.55	2.83	0.21	41
		153.83	10.53	4.61	1.32	25.00	58.55	2.83	0.21	41
		154.50	8.82	4.12	0.00	18.24	68.82	2.21	0.16	20
		155.55	8.33	1.92	0.64	5.13	83.97	1.97	0.11	2
		156.88	7.59	0.69	0.00	9.66	82.07	2.20	0.09	1
		157.57	5.38	0.77	1.54	16.92	75.38	2.13	0.08	8
Muribeca Formation - Oiteirinhos Member	9-GTP-24-SE	426.10	0.55	0.00	1.10	3.30	95.05	0.28	0.03	71
		428.50	2.59	0.00	1.04	5.70	90.67	0.49	0.05	8
		429.05	3.03	0.00	0.00	12.12	84.85	0.62	0.05	45
		432.95	0.00	0.00	0.68	5.48	93.84	0.25	0.00	7
		435.10	1.19	0.00	0.60	1.19	97.02	0.20	0.02	19
		438.05	15.91	11.36	4.55	2.27	65.91	1.09	0.29	242
		438.30	1.12	0.56	1.12	4.47	92.74	0.44	0.03	20
		438.50	13.46	0.00	0.00	23.08	63.46	1.46	0.25	195
		442.55	1.50	0.00	6.00	23.00	69.50	1.37	0.04	0
		447.70	5.94	0.00	0.99	17.82	75.25	0.91	0.08	0
		459.60	5.88	0.49	1.47	36.27	55.88	1.36	0.12	0
		461.50	3.98	0.50	1.99	56.72	36.82	1.13	0.15	0
		465.10	2.75	0.00	2.75	39.56	54.95	1.07	0.07	0
		469.30	6.50	0.00	1.50	31.00	61.00	1.12	0.12	0
		470.60	7.56	0.00	0.58	63.95	27.91	1.12	0.27	0
		474.05	8.50	0.50	2.50	68.00	20.50	1.40	0.42	0
		478.95	1.52	0.00	7.11	48.22	43.15	1.35	0.04	0
		479.53	5.53	0.00	6.53	36.68	51.26	1.65	0.15	0
		483.25	1.58	0.00	3.16	39.47	55.79	1.30	0.04	0
		488.00	2.70	0.00	1.35	27.03	68.92	0.97	0.05	0
		488.65	2.75	0.00	0.00	22.53	74.73	0.92	0.04	0
		492.25	3.21	0.53	2.67	16.04	77.54	1.26	0.07	0
		496.45	0.00	0.00	0.00	8.02	91.98	0.31	0.01	0
		497.43	0.59	0.00	0.00	28.24	71.18	0.66	0.02	0
		501.30	2.55	3.18	5.73	22.29	66.24	1.43	0.11	0
		506.20	0.64	0.00	2.55	24.20	72.61	0.82	0.02	1
		507.45	0.00	0.00	3.23	19.35	77.42	0.63	0.00	0
		509.30	3.07	0.00	3.68	30.06	63.19	1.46	0.09	0
		512.05	4.64	0.66	5.30	24.50	64.90	1.70	0.11	0
		519.18	1.94	0.00	5.83	14.56	77.67	1.52	0.05	0
		527.50	2.37	0.00	2.96	4.73	89.94	0.40	0.05	0
		534.90	0.00	0.00	0.00	0.00	100.00	0.00	0.00	0

		537.65	2.63	0.00	0.00	2.63	94.74	0.24	0.03	0
		547.65	0.00	0.00	0.62	6.83	92.55	0.25	0.00	0
Muribeca Formation – Ibura Member		604.60	0.00	0.00	0.81	2.42	96.77	0.55	0.01	1
		607.35	0.00	0.00	3.85	0.00	96.15	0.00	0.00	1
		613.05	1.63	0.00	0.00	4.07	94.31	0.27	0.03	16
		618.05	6.82	0.00	0.00	0.00	93.18	0.29	0.07	15
		620.53	7.77	0.00	0.00	0.97	91.26	0.54	0.09	18
		623.75	1.19	0.00	0.00	0.00	98.81	0.14	0.01	26
		629.90	1.56	0.00	0.00	0.00	98.44	0.08	0.02	16
		634.95	0.86	0.86	0.00	1.72	96.55	0.35	0.03	0
Muribeca Formation - Oiteirinhos Member	9-GTP-17-SE	497.31	1.16	0.00	0.58	2.91	95.35	0.27	0.02	28
		498.67	1.30	0.00	1.30	2.60	94.81	0.22	0.02	46
		499.50	2.72	0.00	0.54	2.17	94.57	0.31	0.04	16
		501.50	0.00	0.00	0.91	24.55	74.55	0.79	0.01	0
		509.80	0.55	0.00	0.00	8.20	91.26	0.32	0.01	17
		510.40	0.00	0.00	0.50	28.00	71.50	0.65	0.01	0
		511.30	3.00	0.00	0.00	24.00	73.00	0.75	0.04	0
		520.50	5.50	0.00	0.00	9.50	85.00	0.62	0.07	0
		521.50	5.03	0.00	1.01	21.61	72.36	0.97	0.10	0
		532.72	0.00	0.00	0.66	15.79	83.55	0.44	0.00	0
		533.83	1.35	1.35	0.00	27.03	70.27	0.73	0.04	0
		538.30	1.00	0.00	1.00	25.50	72.50	0.66	0.01	0
		539.13	1.00	0.00	0.00	28.00	71.00	0.67	0.02	0
		542.95	0.50	0.00	0.00	26.50	73.00	0.63	0.01	0
		544.15	2.50	0.00	1.00	20.50	76.00	0.77	0.05	0
		548.77	2.01	0.00	1.51	24.12	72.36	0.78	0.03	1
		554.15	1.50	0.00	0.50	22.00	76.00	0.67	0.02	0
		555.27	1.00	0.00	1.00	32.50	65.50	0.81	0.02	0
		556.86	2.00	0.00	0.50	16.50	81.00	0.56	0.02	0
		559.35	1.83	0.00	0.92	9.17	88.07	0.48	0.03	0
		563.25	1.01	0.50	2.01	26.13	70.35	0.82	0.03	1
		566.62	3.50	0.50	0.50	4.00	91.50	0.45	0.05	0
Muribeca Formation – Ibura Member		635.88	2.00	0.00	0.00	3.50	94.50	0.29	0.03	0
		660.65	1.21	0.00	0.00	2.42	96.36	0.20	0.02	7
		663.20	1.46	0.00	0.73	7.30	90.51	0.42	0.02	63
		664.17	5.24	0.00	0.00	9.95	84.82	0.64	0.06	8
		671.40	3.45	0.00	0.00	3.45	93.10	0.30	0.07	54
		676.25	2.66	0.00	0.53	4.79	92.02	0.45	0.05	12
		687.40	5.43	0.00	1.09	19.57	73.91	0.73	0.07	7
		689.25	5.03	0.00	2.51	23.12	69.35	0.95	0.08	1
		690.10	4.50	0.00	0.00	11.00	84.50	0.58	0.06	0
		690.95	5.03	0.00	0.50	19.60	74.87	0.82	0.08	0
Muribeca Formation - Oiteirinhos Member	9-CL-47-SE	330.75	4.00	1.14	2.29	17.71	74.86	2.17	0.06	27
		332.00	8.05	2.68	10.74	38.93	39.60	2.95	0.21	0
		332.67	6.77	1.56	1.56	20.31	69.79	2.50	0.11	0

		333.37	7.65	1.64	1.64	13.66	75.41	2.41	0.11	0
		341.82	7.22	0.52	2.06	22.68	67.53	2.42	0.10	1
		345.40	5.58	0.51	1.52	27.92	64.47	2.47	0.09	0
		347.91	1.61	1.61	2.69	21.51	72.58	2.15	0.04	0
		350.81	3.53	0.59	2.35	15.88	77.65	2.44	0.07	1
		361.80	4.29	3.07	0.61	16.56	75.46	2.15	0.10	17
Muribeca Formation – Ibura Member		572.48	5.63	0.70	2.11	15.49	76.06	2.37	0.08	37
		575.50	2.12	0.00	3.17	11.64	83.07	2.03	0.02	0
		578.55	4.24	0.00	6.06	23.03	66.67	2.56	0.06	0
		578.63	3.59	0.60	1.20	25.15	69.46	2.39	0.06	0
		583.40	6.11	0.00	3.89	20.00	70.00	2.37	0.08	0
		583.70	3.91	0.00	5.03	16.76	74.30	2.25	0.05	1
		590.07	7.10	1.09	6.01	21.86	63.93	2.21	0.12	1
		594.70	6.15	0.56	2.23	12.29	78.77	1.92	0.08	0
		597.20	6.63	0.00	1.10	14.92	77.35	1.93	0.08	0

Table S7.
Percentage abundance of bioclimatic groups and diversity, Fs/X and marine elements for samples from the Araripe Basin. Legend: HG = hygrophytes; HD = hydrophytes; TLF = tropical lowland flora; UF = upland flora; XP = xerophytes; H' = diversity; Fs/X = spores/xerophytes.

Lithostratigraphy	Sections	Depth (m)	HG	HD	TLF	UF	XP	H'	Fs/X	Marine
Romualdo Formation	Sobradinho outcrop (Arai and Assine, 2020)	79.0	6.2	2.1	4.8	32.9	54.1	1.72	0.13	0
		78.7	15.2	3.0	0.0	47.0	34.8	1.56	0.35	0
		78.0	7.4	0.4	0.4	46.8	45.0	1.37	0.15	0
		77.0	18.9	1.0	1.3	49.3	29.5	1.56	0.41	1
		75.5	21.4	1.6	1.4	33.9	41.7	1.70	0.36	0
		75.0	38.1	0.3	0.3	58.2	3.1	1.52	0.93	0
		74.6	4.0	0.0	0.0	14.0	82.0	0.80	0.07	0
		73.0	11.5	5.1	5.1	17.9	60.3	1.88	0.25	3
		72.0	14.3	2.9	2.9	54.3	25.7	2.19	0.40	0
		70.0	4.8	3.4	4.1	55.1	32.7	1.45	0.20	0
		68.0	11.1	11.1	5.6	27.8	44.4	1.74	0.33	0
		67.8	13.0	8.7	0.0	43.5	34.8	1.57	0.38	0
		66.0	37.1	9.7	1.6	14.5	37.1	2.07	0.57	0
		65.0	8.6	6.9	3.4	16.4	64.7	1.44	0.21	0
		64.2	4.7	2.3	0.0	18.6	74.4	1.14	0.16	0
		63.8	68.8	6.3	0.0	18.8	6.3	1.67	0.92	0
		63.4	25.5	3.4	2.0	8.7	60.4	1.83	0.34	0
		59.2	13.6	0.6	0.0	28.4	57.4	1.46	0.21	0
		57.4	13.8	0.6	2.8	6.1	76.8	1.20	0.17	7
		57.0	4.1	2.0	2.9	6.9	84.1	0.91	0.07	79
		56.0	13.6	0.0	3.9	20.1	62.3	1.52	0.18	9
		53.1	0.0	0.0	0.0	6.3	93.8	0.91	0.06	0
		50.0	11.2	3.4	4.5	3.4	77.5	1.43	0.19	1
		46.0	22.4	2.2	6.0	3.7	65.7	1.77	0.29	2
		45.7	14.3	0.0	0.0	3.6	82.1	1.28	0.15	4
		45.2	0.0	2.1	0.0	10.4	87.5	0.68	0.05	0
		44.8	12.8	0.0	0.0	6.0	81.2	0.91	0.14	0
		43.0	5.6	2.1	4.2	7.0	81.1	0.88	0.10	0
		42.3	9.1	0.0	0.0	0.0	90.9	0.30	0.09	0
		41.1	5.1	0.0	0.0	2.6	92.3	0.47	0.05	0
		38.4	4.0	0.0	0.0	0.0	96.0	0.17	0.04	0
		37.4	2.7	0.0	0.0	1.4	95.9	0.29	0.03	0
		36.1	8.3	2.8	0.0	5.6	83.3	0.62	0.12	2
		35.0	7.7	1.5	1.5	9.2	80.0	1.02	0.13	20
		34.7	0.0	0.0	1.2	6.0	92.8	0.44	0.00	0
		34.0	5.8	0.0	3.8	13.5	76.9	0.91	0.13	0
		33.5	3.9	0.0	3.2	7.1	85.8	0.94	0.05	0
		31.0	47.3	21.8	1.8	14.5	14.5	2.28	0.85	1

	1-PS-11-CE (Rios-Netto, 2011)	4.4	13.2	7.0	3.1	37.7	38.9	2.12	0.52	3021
		10.0	6.6	8.8	2.6	68.4	13.6	1.64	0.74	251
		12.4	5.4	6.9	4.5	62.2	20.8	1.83	0.54	225
		15.0	39.6	8.3	1.5	46.9	3.7	1.61	0.95	200
		17.4	8.7	13.0	4.0	46.8	27.4	2.17	0.54	51
		20.0	9.8	24.9	1.8	51.6	12.0	1.83	0.82	1
		53.1	9.4	1.8	0.0	85.4	3.5	1.23	0.93	0
	2-AP-1-CE	396.3	0.0	0.0	2.9	0.0	97.1	0.26	0.00	0
		396.5	0.0	0.0	0.0	1.3	98.7	0.07	0.00	4
		396.8	0.0	0.0	0.0	0.0	100.0	0.00	0.05	5
		397.2	3.4	3.4	0.0	3.4	89.7	0.49	0.07	5
		397.7	4.2	0.8	0.3	2.6	92.2	0.46	0.05	0
		398.1	0.0	0.0	0.0	4.2	95.8	0.62	0.00	6
		398.4	0.0	0.0	0.0	2.9	97.1	0.55	0.00	16
		398.6	0.0	0.0	8.7	4.3	87.0	0.53	0.00	2
		399.3	5.0	0.0	5.0	0.0	90.0	0.78	0.05	3
Ipubi Formation	1-PS-11-CE (Rios-Netto, 2011)	54.4	0.0	0.0	0.0	5.7	94.3	0.81	0.04	0
		55.2	1.3	0.0	1.3	5.1	92.3	0.87	0.06	0
		57.0	3.5	0.5	1.6	21.2	73.2	1.34	0.11	0
		58.0	0.0	0.0	0.0	24.3	75.7	0.84	0.13	0
		59.3	4.8	0.0	0.0	9.5	85.7	0.85	0.12	0
		60.8	3.0	1.5	0.0	13.4	82.1	1.17	0.08	9
		62.5	14.3	0.0	28.6	14.3	42.9	1.44	0.40	0
		63.3	12.7	2.5	0.0	56.1	28.7	1.78	0.58	0
		64.5	33.3	0.0	0.0	25.0	41.7	1.58	0.55	1
		66.0	0.0	6.7	0.0	46.7	46.7	1.08	0.50	0
		67.0	5.1	0.6	1.8	12.8	79.8	1.38	0.10	0
		69.4	5.5	1.1	1.3	30.6	61.5	1.42	0.16	0
Crato Formation	1-PS-11-CE (Rios-Netto, 2011)	77.0	9.1	9.1	4.5	22.7	54.5	1.31	0.18	0
		79.6	3.2	4.2	1.2	40.5	51.0	1.82	0.28	0
		86.6	5.4	0.0	0.6	12.9	81.1	0.83	0.08	0
		88.0	14.8	0.0	0.9	38.0	46.3	1.91	0.42	0
		92.0	3.3	0.0	0.4	26.4	69.9	1.47	0.17	0
		93.2	0.4	1.8	49.5	18.6	29.7	1.80	0.32	0
		113.6	5.9	0.4	2.2	64.2	27.3	1.71	0.52	1
		123.5	8.3	0.6	0.6	71.8	18.8	1.57	0.76	0
		125.8	18.2	0.0	2.3	18.9	60.6	1.65	0.32	0
		128.9	8.5	0.0	0.0	74.4	17.1	1.38	0.67	0
		130.3	5.7	0.5	3.1	77.1	13.5	1.45	0.85	0
		137.3	2.7	3.6	4.5	70.3	18.9	1.76	0.73	0
		138.8	9.5	0.0	0.0	90.5	0.0	0.31	1.00	0
		143.0	13.0	0.0	0.0	70.4	16.7	1.36	0.82	0
		145.1	6.8	0.7	1.4	83.1	8.1	1.37	0.87	0
		147.5	10.7	3.3	9.8	54.2	22.0	2.11	0.62	5
		150.6	3.6	0.0	7.7	81.0	7.7	1.48	0.88	0

		153.7	30.5	8.5	10.2	35.6	15.3	2.01	0.80	0
		159.7	40.9	0.0	0.0	52.3	6.8	1.06	0.93	0
	2-AP-1-CE	452.8	4.5	0.0	11.4	13.6	70.5	1.61	0.11	4
		453.2	2.6	0.0	0.0	0.9	96.5	0.63	0.03	1
		454.3	3.3	0.0	3.3	10.0	83.3	1.04	0.04	4
		455.6	7.7	1.4	0.0	2.8	88.0	0.90	0.11	1
		456.2	2.2	0.7	0.7	0.7	95.7	0.34	0.04	2
		456.7	43.1	7.2	0.6	0.6	48.5	1.41	0.51	1
		486.8	7.7	1.3	18.1	0.0	72.9	1.32	0.11	0
		488.1	25.0	0.0	0.0	62.5	12.5	1.91	0.80	0

Table S8.
Percentage abundance of bioclimatic groups and diversity, Fs/X and marine elements for samples from the Espírito Santo Basin. Legend: HG = hygrophytes; HD = hydrophytes; TLF = tropical lowland flora; UF = upland flora; XP = xerophytes; H' = diversity; Fs/X = spores/xerophytes.

Lithostratigraphy	Sections	Depth (m)	HG	HD	TLF	UF	XP	H'	Fs/X	Marine
Regência Formation	9-PEI-3-ES	889.87	1.0	0.0	0.7	0.7	97.6	0.15	0.01	3
		891.72	2.4	7.7	1.2	0.6	88.1	0.49	0.10	3
		896.6	3.3	0.0	1.3	1.3	94.0	0.38	0.03	5
		899.02	3.5	0.4	0.9	0.4	94.8	0.22	0.04	15
São Mateus Formation		971.3	9.5	0.0	11.8	3.0	75.7	0.99	0.11	0
Mariricu Formation - Itaúnas Member		972.5	11.6	0.4	6.9	1.8	79.3	0.84	0.13	0
		979.29	7.4	0.0	2.0	1.3	89.3	0.46	0.08	4
		980.1	5.2	0.0	6.2	1.0	87.5	0.54	0.06	1
		980.57	2.9	0.0	8.2	1.6	87.3	0.58	0.03	4
		981.1	3.7	0.0	6.1	0.7	89.6	0.56	0.04	3
		985.35	13.8	0.0	6.2	3.1	76.9	0.85	0.15	0
Regência Formation	9-PEI-6-ES	569.16	6.1	0.0	1.0	0.0	92.9	0.24	0.06	6
		662.14	9.0	4.0	6.0	3.0	77.9	0.86	0.14	0
		662.92	6.7	0.0	7.7	3.7	81.9	0.55	0.08	0
		662.92	5.0	0.0	8.7	5.0	81.2	0.78	0.06	1
		663.25	2.0	0.0	12.0	2.7	83.4	0.69	0.02	1
		664.6	5.8	0.0	4.1	0.7	89.5	0.76	0.06	2
		664.93	3.7	0.0	4.3	0.7	91.3	0.50	0.04	2
		665.58	3.6	0.0	4.0	2.0	90.4	0.64	0.04	45
		665.8	2.5	0.0	4.6	0.7	92.2	0.59	0.03	18
Mariricu Formation - Itaúnas Member		688.6	29.1	0.0	0.4	1.1	69.3	0.78	0.30	0
		746.9	0.0	0.0	2.9	0.0	97.1	1.04	0.00	0
		748.1	7.8	0.0	4.5	1.0	86.7	1.11	0.08	0
		748.9	3.3	0.0	1.1	0.7	94.8	0.52	0.03	0
		754.3	13.2	0.0	2.6	7.9	76.3	0.73	0.15	0

Table S9.

Average abundance of bioclimatic groups, diversity, Fs/X and marine elements of the paleoclimatic phases for the Bragança-Viseu, São Luís and Parnaíba basins. No marine elements.

Paleoclimatic phases	Hygrophytes	Hydrophytes	Tropical lowland flora	Upland flora	Xerophytes	Diversity (H’)	Fs/X	Indval
Pre-evaporitic	18.8	0.7	5.6	14.1	60.7	2.0	0.3	*Deltoidospora* sp. (80.6)
Evaporitic	10.0	1.0	16.0	5.0	67.9	2.2	0.1	*Afropollis* spp. (79.3%)
Post-evaporitic	15.5	0.6	14.4	22.0	47.4	2.1	0.3	*Deltoidospora* sp. (86.2%)
General average of the B.-V, S.L. and Parnaíba	14.8	0.8	12.0	13.7	58.7	2.1	0.2	
General average	15.6	1.4	3.1	19.5	60.5	1.4	0.3	

Table S10.

Average abundance of bioclimatic groups, diversity, Fs/X and marine elements of the paleoclimatic phases for the Ceará Basin.

Paleoclimatic phases	Hygrophytes	Hydrophytes	Tropical lowland flora	Upland flora	Xerophytes	Diversity (H’)	Fs/X	Marine	IndVal
Pre-evaporitic	16.7	5.1	3.3	17.5	57.4	2.5	0.3	0.0	*Deltoidospora* spp. (100%)
Evaporitic	15.4	0.7	3.8	14.0	66.0	1.6	0.2	0.0	*Classopollis*.spp. (99.6%)
Post-evaporitic	27.4	0.7	3.0	33.7	35.3	2.9	0.4	2.0	*Araucariacites limbatus* (100%)
General average of the Ceará Basin	19.8	2.2	3.4	21.7	52.9	2.3	0.3	0.7	
General average	15.6	1.4	3.1	19.5	60.5	1.4	0.3	16.1	

Table S11.

Average abundance of bioclimatic groups, diversity, Fs/X and marine elements of the paleoclimatic phases for the Potiguar Basin.

Paleoclimatic phases	Hygrophytes	Hydrophytes	Tropical lowland flora	Upland flora	Xerophytes	Diversity (H’)	Fs/X	Marine	IndVal
Pre-evaporitic	29.6	4.8	0.9	36.5	28.2	2.0	0.6	-	*Gnetaceaepollenites* spp. (74,1%)
Evaporitic	7.7	0.2	0.5	11.7	79.9	0.7	0.1	-	*Classopollis* spp. (56,4%)
Post-evaporitic	10.1	0.1	3.0	20.5	66.3	1.4	0.2	2.0	*Cicatricosisporites* spp. (60,6%)
General average of the Potiguar Basin	24.1	3.6	1.2	31.0	40.2	1.7	0.4	0.3	
General average	15.6	1.4	3.1	19.5	60.5	1.4	0.3	16.1	

Table S12.

Average abundance of bioclimatic groups, diversity, Fs/X and marine elements of the paleoclimatic phases for the Sergipe Basin.

Paleoclimatic phases	Hygrophytes	Hydrophytes	Tropical lowland flora	Upland flora	Xerophytes	Diversity (H')	Fs/X	Marine	IndVal
Evaporitic	3.8	0.1	1.5	10.2	84.4	1.0	0.1	10.5	*Classopollis*. *classoides* (74.9%)
Post-evaporitic	11.5	0.8	1.5	26.0	60.2	1.3	0.2	42.61	*Cicatricosisporites sp.* (52,0%) *Araucariacites australis* (50,4%)
General average of the Sergipe Basin	7.7	0.5	1.5	18.1	72.3	1.2	0.2	26.6	
General average	15.6	1.4	3.1	19.5	60.5	1.4	0.3	16.1	

Table S13.

Average abundance of bioclimatic groups, diversity, Fs/X and marine elements of the paleoclimatic phases for the Araripe Basin.

Paleoclimatic phases	Hygrophytes	Hydrophytes	Tropical lowland flora	Upland flora	Xerophytes	Diversity (H')	Fs/X	Marine	IndVal
Pre-evaporitic	34.6	0.9	5.5	17.4	41.6	1.1	0.5	0.7	*Equisetosporites* spp. (51,7%)
Evaporitic	7.0	1.1	2.9	22.1	67.0	1.2	0.2	0.8	*Classopollis intrareticulatus* (34,4%)
Post-evaporitic	11.5	3.4	1.9	21.1	62.2	1.2	0.3	73.4	*Inaperturopollenites* spp. (48,6%)
General average of the Araripe Basin	17.7	1.8	3.4	20.2	56.9	1.2	0.3	25.0	
General average	15.6	1.4	3.1	19.5	60.5	1.4	0.3	16.1	

Table S14.

Average abundance of bioclimatic groups, diversity, Fs/X and marine elements of the paleoclimatic phases for the Espírito Santo Basin.

Paleoclimatic phases	Hygrophytes	Hydrophytes	Tropical lowland flora	Upland flora	Xerophytes	Diversity (H')	Fs/X	Marine	IndVal
Evaporites	8.9	0	4.3	1.8	84.9	0.7	0.1	1.1	*Gnetaceaepollenites* spp. (45,7%)
Post-evaporites	4.6	0.9	4.9	1.7	87.9	0.6	0.1	7.2	*Inaperturopollenites simplex* (68,8%)
General average of the Espírito Santo Basin	6.8	0.5	4.6	1.8	86.4	0.7	0.1	4.2	
General average	15.6	1.4	3.1	19.5	60.5	1.4	0.3	16.1	

Table S15.
Indicator species values (IndVal) in relation to paleoclimatic phases for the individual basins.

Paleoclimatic phases	**B.-V, S.L. and Parnaíba basins**	**Ceará Basin**	**Potiguar Basin**	**Sergipe Basin**	**Araripe Basin**	**Espírito Santo Basin**
Post-evaporitic	*Deltoidospora* spp. (86.2%)	*Araucariacites limbatus* (100%)	*Cicatricosisporites* spp. (60.6%)	*Cicatricosisporites* spp. (52.0%) *Araucariacites autralis* (50.4%)	*Inaperturopollenites* spp. (48.6%)	*Inaperturopollenites simplex* (68.8%)
Evaporitic	*Afropollis* spp. (79.3%)	*Classopollis* spp. (99.6%)	*Classopollis* spp. (56.4%)	*Classopollis classoides* (74.9%)	*Classopollis intrareticulatus* (34.4%)	*Gnetaceaepollenites* spp. (45.7%)
Pre-evaporitic	*Deltoidospora* spp. (80.6%)	*Deltoidospora*.sp. (100%)	*Gnetaceaepollenites* spp. (74.1%)	-	*Equisetosporites* spp. (51.7%)	-
